# Genetic dissection of climacteric fruit ripening in a melon population segregating for ripening behavior

**DOI:** 10.1038/s41438-020-00411-z

**Published:** 2020-11-01

**Authors:** Lara Pereira, Miguel Santo Domingo, Valentino Ruggieri, Jason Argyris, Michael A. Phillips, Guangwei Zhao, Qun Lian, Yongyang Xu, Yuhua He, Sanwen Huang, Marta Pujol, Jordi Garcia-Mas

**Affiliations:** 1 Centre for Research in Agricultural Genomics (CRAG) CSIC-IRTA-UAB-UB, Edifici CRAG, Campus UAB, 08193 Cerdanyola, Barcelona Spain; 2 IRTA (Institut de Recerca i Tecnologia Agroalimentàries), Edifici CRAG, Campus UAB, 08193 Cerdanyola, Barcelona Spain; 3 Department of Biology, University of Toronto–Mississauga, Mississauga, ON L5L 1C6 Canada; 4 Zhengzhou Fruit Research Institute, Chinese Academy of Agricultural Sciences, Zhengzhou, China; 5 Shenzhen Branch, Guangdong Laboratory for Lingnan Modern Agriculture, Genome Analysis Laboratory of the Ministry of Agriculture, Agricultural Genomics Institute at Shenzhen, Chinese Academy of Agricultural Sciences, Shenzhen, China

**Keywords:** Plant breeding, Plant genetics

## Abstract

Melon is as an alternative model to understand fruit ripening due to the coexistence of climacteric and non-climacteric varieties within the same species, allowing the study of the processes that regulate this complex trait with genetic approaches. We phenotyped a population of recombinant inbred lines (RILs), obtained by crossing a climacteric (Védrantais, cantalupensis type) and a non-climcteric variety (Piel de Sapo T111, inodorus type), for traits related to climacteric maturation and ethylene production. Individuals in the RIL population exhibited various combinations of phenotypes that differed in the amount of ethylene produced, the early onset of ethylene production, and other phenotypes associated with ripening. We characterized a major QTL on chromosome 8, *ETHQV8.1*, which is sufficient to activate climacteric ripening, and other minor QTLs that may modulate the climacteric response. The *ETHQV8.1* allele was validated by using two reciprocal introgression line populations generated by crossing Védrantais and Piel de Sapo and analyzing the *ETHQV8.1* region in each of the genetic backgrounds. A Genome-wide association study (GWAS) using 211 accessions of the ssp. *melo* further identified two regions on chromosome 8 associated with the production of aromas, one of these regions overlapping with the 154.1 kb interval containing *ETHQV8.1*. The *ETHQV8.1* region contains several candidate genes that may be related to fruit ripening. This work sheds light into the regulation mechanisms of a complex trait such as fruit ripening.

## Introduction

Fleshy fruits are an important component of human diet and a major source of nutritional compounds. The ultimate biological function of fruit is for seed dispersal. Once fruit growth has been accomplished, fleshy fruits undergo a series of physiological and metabolic changes to become edible and attractive to animals^1^. Among these changes; sugar and organic acid accumulation, decay of fruit firmness, color change and synthesis of volatiles commonly occur. Fleshy fruits are classified into two physiological groups depending on the involvement of the hormone ethylene during ripening: climacteric fruits show a transient rise in respiration accompanied by the autocatalytic synthesis of ethylene at the onset of ripening, leading to a peak of this hormone that, in contrast, is absent in non-climacteric fruits^2^. It has been suggested that there is a partial overlap between both types of ripening^3,4^.

In climacteric fruits, the presence of ethylene is necessary to trigger multiple responses, among them a change of texture, principally softening of mesocarp, mediated by cell wall degrading enzymes; accumulation of pigments such as flavonoids and carotenoids in parallel with chlorophyll degradation, leading to changes in rind and flesh color, synthesis of sugars, organic acids and a diverse array of volatiles, allowing the fruit to reach an attractive flavor and taste, and the formation of an abscission layer in the pedicel^5^. Tomato is considered the biological model to understand climacteric fruit ripening. Two enzymes of the biosynthetic pathway of ethylene, 1-aminocyclopropane-1-carboxylate (ACC) synthase and ACC oxidase, are regulated in a fine and complex way by transcription factors (TFs) as *RIN*^6^, *NOR*^5^, and *CNR*^7^. In addition, other TFs modulating the initiation or evolution of fruit ripening have been characterized, as *FUL1/FUL2*^3,8^, *AP2a*^9^, *GLK2*^10^, *TAGL1*^11^, and *HB-1*^12^, among others. Mutations or overexpression of these TFs influence ethylene production and some of its downstream effects, such as accumulation of carotenoids or changes in texture. Recently, several studies have proved that DNA demethylation has a major influence in controlling the onset of fruit ripening^13–15^. In mutants for some of the TFs described above, several ripening responses are compromised, giving rise to unripe fruits or fruits with a substantial delay in ripening when compared to wild type fruits. Most of these processes found in climacteric fruits are successfully achieved in non-climacteric fruits, probably through a combination of common mechanisms^16^ and different hormonal pathways^17^.

Although many advances have been achieved in understanding climacteric ripening in tomato, knowledge of the fruit ripening process in other crops is less well-understood^18–20^. Melon (*Cucumis melo* L.) has been proposed as an alternative model to understand fruit ripening due to the coexistence of climacteric and non-climacteric varieties within the same species^21^. Melons show a wide range in the degree of climacteric response, from the maximally climacteric varieties belonging to the *cantalupensis* group as “Védrantais” (Ved) to the non-climacteric varieties from the *inodorus* group as “Piel de Sapo” (PS), which undergo virtually none^22^. The changes promoted by ethylene during melon fruit ripening are slightly different to those described in tomato. Silencing of *Aco1* in a Ved background allowed the identification of ethylene-dependent ripening processes^23^. Flesh carotenoid and sugar accumulation, acidity and, partially, flesh softening are, unlike in tomato, ethylene-independent in melon^24^. QTLs controlling climacteric ripening and ethylene production have been described in segregating populations obtained from melon accessions showing different ripening behaviors^25–27^. So far only one of these QTLs, *ETHQV6.3*, has been cloned. An introgression of the non-climacteric accession PI 161375, containing *ETHQV6.3*, into the non-climacteric PS induced climacteric ripening^26^. The *ETHQV6.3* underlying gene is *CmNAC-NOR*, the orthologue of the tomato *NOR*^28^, suggesting that common mechanisms are involved in climacteric ripening control in both species.

A recombinant inbred line (RIL) population from a cross between Ved and PS was developed and utilized to map QTLs related to fruit quality and morphology^29^. The aim of this work was to characterize ethylene production and climacteric ripening behavior in this RIL population, which was also segregating for the climacteric behavior, and to identify additional genetic factors that regulate this process.

## Results

### Ethylene production and climacteric ripening in the Ved × PS RIL population

**Fig. 1:**
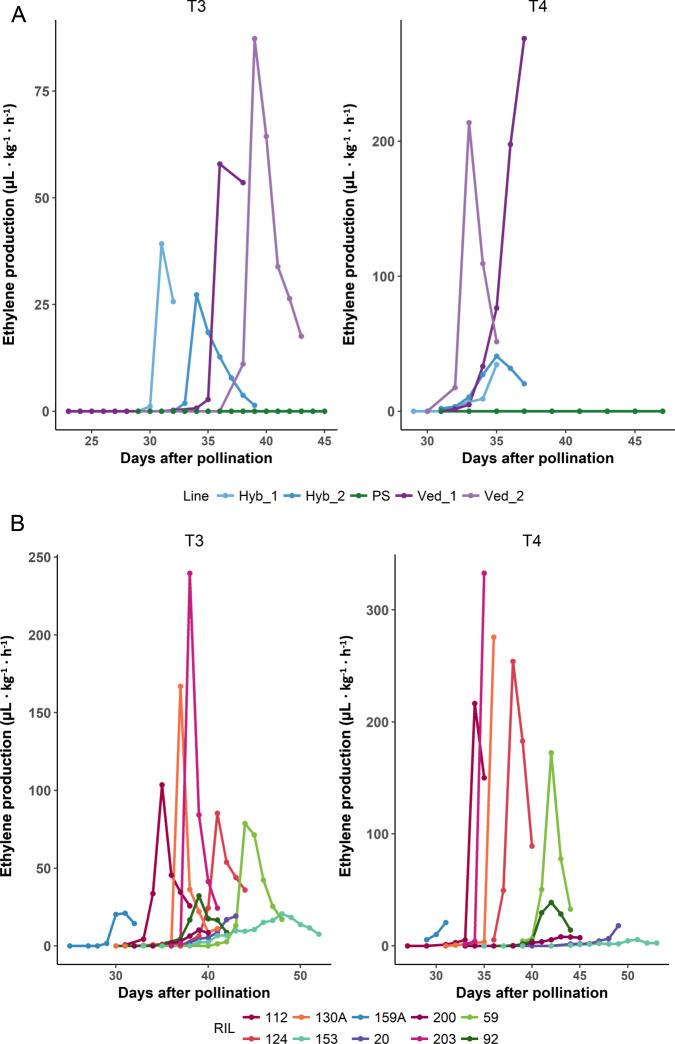
Ethylene production in *planta*. **A** Ethylene emission rates in Ved, PS and Hyb attached fruits in T3 and T4 blocks. **B** Ethylene emission in a representative subset of RILs in T3 and T4 blocks

The RIL population parents were Ved, a highly climacteric *cantalupensis* line, and PS, a non-climacteric *inodorus* type. Several ripening-related traits were measured in both parents (see “Methods” section and Supplementary Table 1). Ved showed maximal climacteric behavior starting around 34 days after pollination (DAP) in all blocks: strong sweet aroma (assessed subjectively by smelling the fruit), slight change of color from white to cream, and abscission layer formation with fruit abscission in most cases (Supplementary Table 1). PS did not show any symptom of climacteric ripening in any block. The hybrid line (Hyb) presented an intermediate phenotype between the parents, being always climacteric but showing some of the phenotypic effects later than Ved; however, the flesh firmness in Hyb fruits was lower than either parents. These phenotypes correlate with the measured ethylene production levels: Ved produced a high amount of ethylene (72.6 and 224.9 µL kg^−1^ h^−1^ in T3 and T4, respectively), PS did not produce ethylene, and Hyb showed an intermediate peak (33.3 and 37.7 µL kg^−1^ h^−1^ in T3 and T4, respectively) (Fig. 1A). Although this pattern was consistent over two years, we observed that ethylene production (ETH) and, to a lesser extent, earliness of ethylene production (DAPE) and earliness of ethylene peak (DAPP), are probably environment-dependent.

The statistics for climacteric traits in the RIL population are presented in Table 1, where segregation was observed for all of them. Ethylene emission from fruit of a representative subset of RILs was measured over time, revealing a transient peak of production (Fig. 1B). Data for both years were treated separately due to the effect of environment on ethylene peak level and earliness; however, the general pattern was very similar. Earliness, height, and shape of the ethylene peak segregated in the RIL population. We could observe an independent segregation, at least partially, between ETH and DAPE/DAPP. For example, RIL 159A presented an early ethylene peak but the maximum amount of ethylene was low in comparison with other lines; on the opposite side, RIL 59 showed a considerably high ethylene peak around 43 DAP, with a delay of 8–9 days compared to Ved (Fig. 1A, B). Transgressive segregation was observed for ETH in some RILs, which was consistent between years, as well as for DAPP and DAPE.

**Table 1:** Basic statistics for climacteric ripening traits and ethylene production in the RIL population

Trait (units)	Code	Mean	SD	Median	Range
Production of aroma	ARO	0.69	0.34	0.75	0–1
Earliness of production of aroma (DAP)	EARO	43.05	6.68	41.67	30.6–60
Chlorophyll degradation	CD	0.46	0.38	0.50	0–1
Earliness of chlorophyll degradation (DAP)	ECD	41.39	6.55	40.00	27.5–58
Earliness of abscission layer formation (DAP)	EALF	43.93	7.32	42.50	28–60
Abscission	ABS	1.25	1.04	1.00	0–3
Harvest date (DAP)	HAR	51.07	7.82	50.80	29.5–62
Flesh firmness (kg cm^−2^)	FIR	3.05	1.62	2.80	0.74–7.14
Maximum ethylene production (µL eth kg^−1^ h^−1^)	ETH	31.05	52.69	13.03	0–286.22
Earliness of ethylene production (DAP)	DAPE	38.23	5.18	37.50	27.5–50
Earliness of ethylene peak (DAP)	DAPP	42.21	5.89	42.00	32–58
Width of ethylene peak (days)	WEP	3.71	1.82	3.50	0–9

**Fig. 2:**
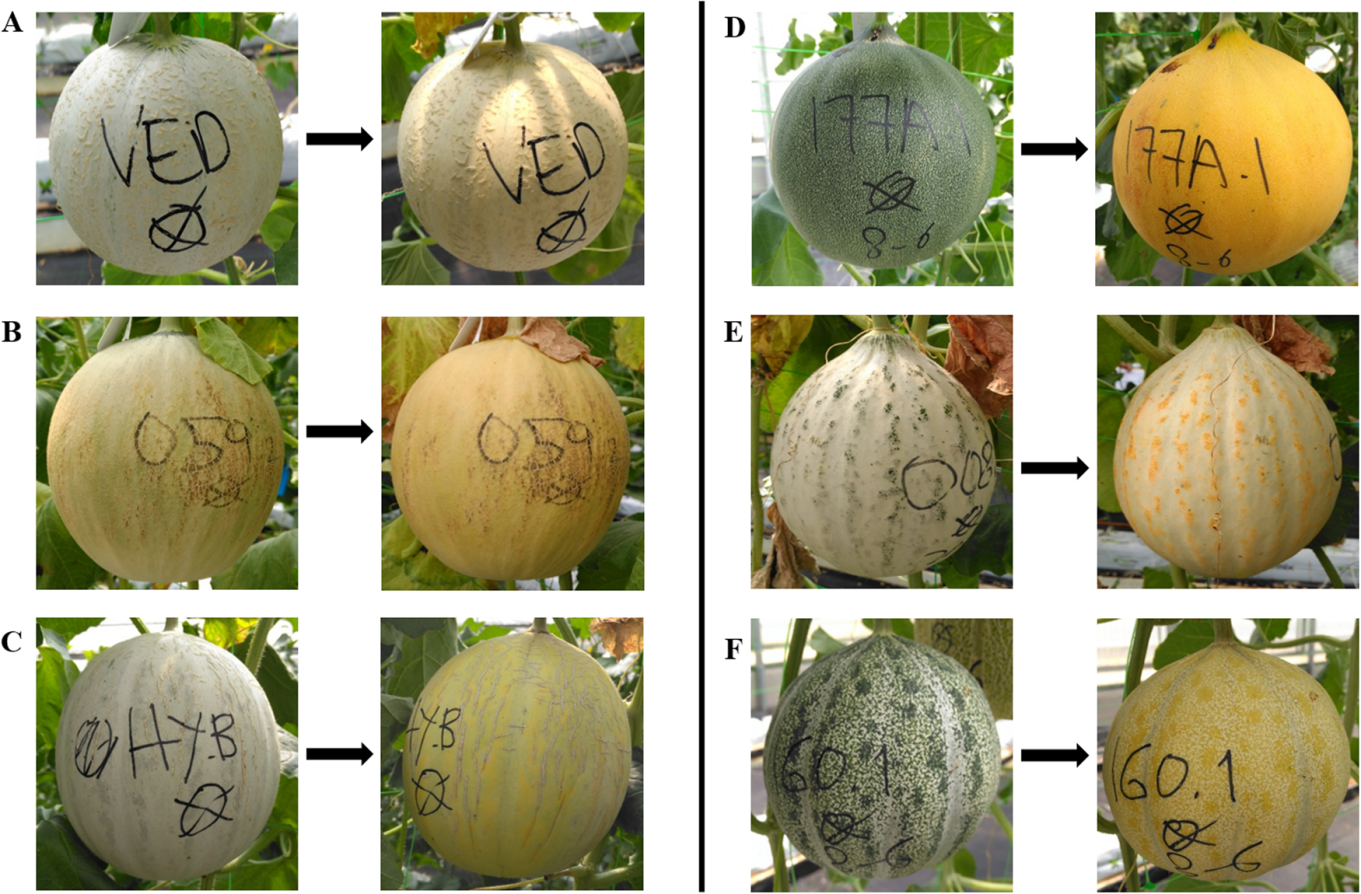
Immature (left) and ripe (right) fruit rind for Ved, Hyb, and several RILs. **A** Ved. **B** RIL59. **C** Hybrid (Hyb). **D** RIL177A. **E** RIL8. **F** RIL160

Most of the RILs produced aromatic fruits that formed abscission layers, but only half of them changed their rind color during ripening. The change of color in the fruit rind, mainly attributable to chlorophyll degradation, manifested itself differently depending on the line (Fig. 2). Some melons, with white rind when immature, turned to a cream-slight orange color when ripe (Fig. 2A, B); the color change in these cases was subtle and the clearest effect was observed in the region near the pedicel, where green color turned to yellow or slight orange. The Hyb clearly turned from white rind to orange-yellow when ripe (Fig. 2C). Other fruits were green when immature and during the ripening process became bright yellow (Fig. 2D, F). In some cases, we observed a striking pattern in mottled rinds, where the spots changed color when ripe (Fig. 2E, F). Concerning the abscission layer formation, the RIL population included lines that did not abscise (Supplementary Fig. 1A), lines with complete fruit abscission (Supplementary Fig. 1D) and intermediate phenotypes with subtle and/or partial abscission layer formation (Supplementary Fig. 1B) and marked scar formation in the abscission zone (Supplementary Fig. 1C). Only 31% of the climacteric lines presenting abscission layer fell from the plant, and even the controls Ved and Hyb did not always show abscission, which, according to our results, seems to be highly dependent on environmental factors as temperature and light.

Generally, ethylene production is measured in detached fruits, precluding the precise phenotyping of other ripening-related traits. The method used in our study allows the fruit to follow the physiological process of ripening without any alterations. Therefore the phenotyping of downstream effects of ethylene can be observed non-invasively in planta. The most common, and generally earliest climacteric symptom was sweet aroma production, which appeared on average four days after the starting of DAPE and in many RILs before DAPP. When a change of rind color was induced, it appeared almost simultaneously with ethylene production; the first or second day of detectable ethylene production, chlorophyll degradation started to be appreciable and in approximately three days the color change was complete. Commonly, abscission layer formation was the last trait exhibited, around five days after ethylene detection. However, we could observe that depending on the RIL, the earliness and the penetrance of the phenotype varied, with some lines that fell from the plant on the first or second day of ethylene production and others that remained with a subtle abscission layer for weeks.

Surprisingly, the correlation between ETH and the appearance and earliness of climacteric symptoms was not high (Supplementary Fig. 2). Earliness of production of aroma (EARO), DAPE, DAPP, and harvest date (HAR) were highly and positively correlated, in agreement with our observations. Production of aroma (ARO) was highly correlated with all the other phenotypic effects, positively with abscission (ABS) and chlorophyll degradation (CD) and negatively with flesh firmness (FIR) and with earliness of ripening (DAPP, DAPE, earliness of production of aroma (EARO), earliness of chlorophyll degradation (ECD), HAR), but slightly correlated to ETH.

### QTL mapping

**Fig. 3:**
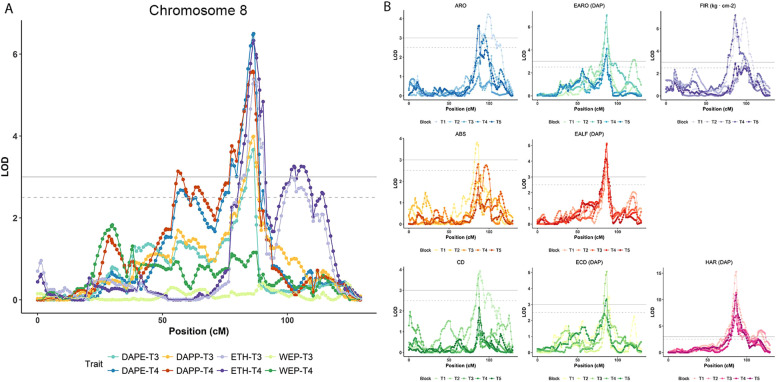
A major *QTL ETHQV8.1* mapped in chromosome 8. **A** For maximum ethylene production (ETH), earliness of ethylene production (DAPE), width of ethylene peak (WEP) and earliness of ethylene peak (DAPP) in T3 and T4. **B** for the rest of climacteric traits

We performed two complementary mapping experiments, the first one for each individual block independently and the second one using the mean of the phenotyped blocks for each RIL. In the first experiment, we mapped 74 QTLs for all traits on almost all melon chromosomes (Supplementary Table 2A). The QTLs are represented in Fig. 3 and Supplementary Fig. 3, indicating for each trait the LOD scores and the genetic position across the chromosomes.

A major QTL, *ETHQV8.1*, was mapped in chromosome 8, with a maximum LOD around the position 86.49 cM. *ETHQV8.1* was detected for ETH, DAPE and DAPP in both years with significant LOD scores (e.g. 6.33 for ETH in T4 (Fig. 3A)). Other traits associated to the earliness of climacteric ripening, such as EARO, earliness of abscission layer formation (EALF) and HAR, also showed consistent significant QTLs in this region (Fig. 3B). Traits more related to the presence/absence of climacteric ripening, as ARO, CD, and ABS, presented significant LOD scores only in some of the blocks. In all cases, the Ved allele underlying *ETHQV8.1* intensified the climacteric effect, meaning higher and earlier ethylene production as well as earlier aroma production, chlorophyll degradation, and abscission layer formation. The impact of the QTL was more obvious for the earliness of climacteric symptoms rather than for their presence.

Other minor QTLs were characterized in chromosomes 2, 3, 6, 7, 10, and 11. Ethylene production QTLs were only found in chromosomes 2 and 11. In some cases, QTLs were found for only one of the climacteric traits: the QTL in the center of chromosome 6 affecting mostly CD and the QTL in the top of chromosome 10 influencing only FIR.

**Fig. 4:**
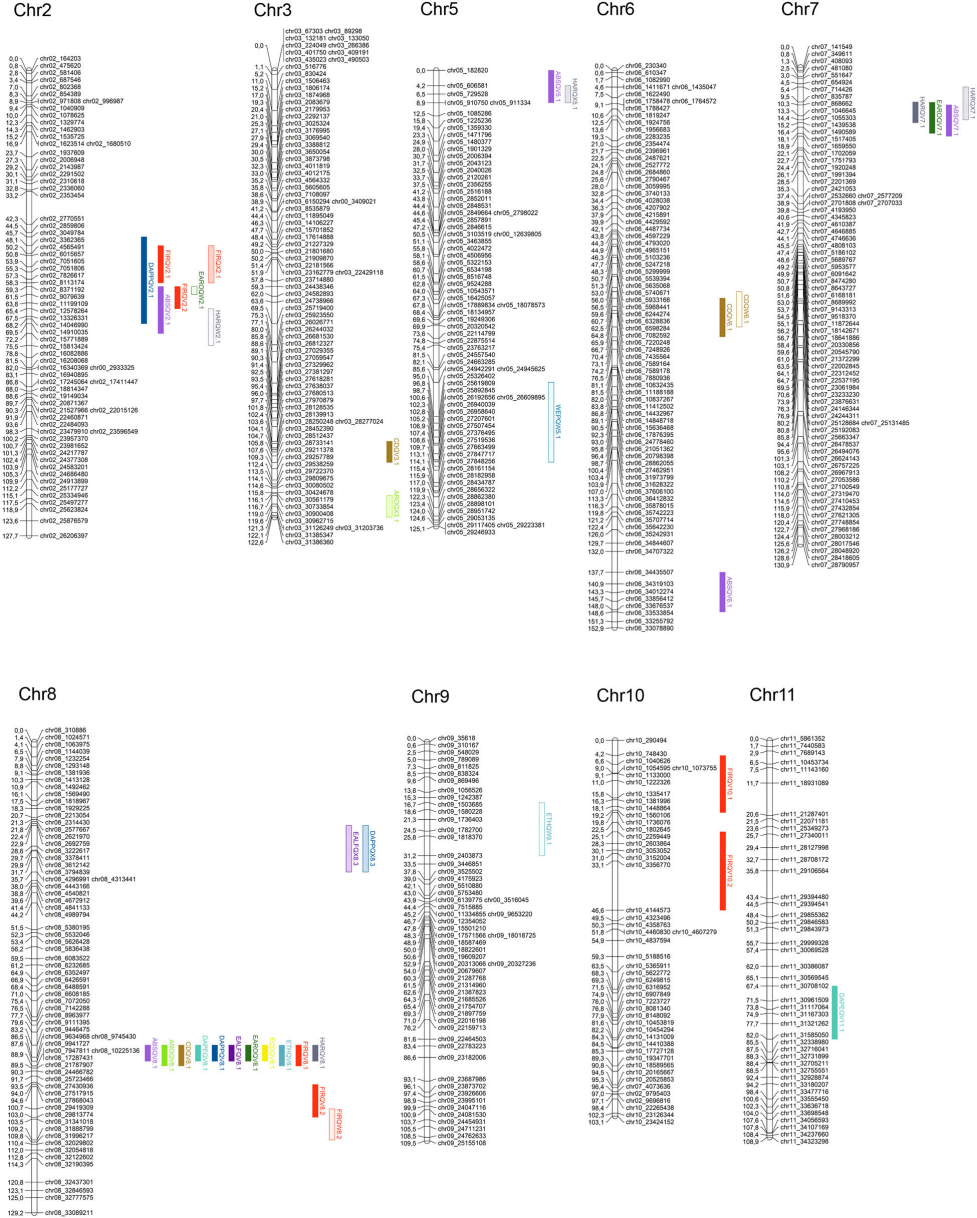
QTL mapping analysis performed using the mean values of the phenotyped blocks. Only chromosomes containing QTL are displayed. Each color corresponds to a different trait: ARO, light green; EARO, dark green; CD, brown; ECD, yellow; EALF, dark purple; ABS, light purple; HAR, grey; FIR, red; ETH, light blue; DAPE, turquoise; DAPP, dark blue; WEP, blue

In the second mapping experiment we performed a QTL analysis with the mean values of the phenotyped blocks. The results (Table 2 and Fig. 4) overlap to a high extent with the first mapping experiment, but with fewer QTL^26^ detected. *ETHQV8.1* was detected for all evaluated traits, except for width of ethylene peak (WEP), with LOD > 3, allowing to delimit a confidence interval of 4.3 cM spanning around 500 kb, with a maximum LOD score in the physical position 9,634,968 bp. In all cases, the Ved allele intensified the climacteric behavior, and an additive effect as high as 29.7 µL ethylene kg^−1^ h^−1^ in ETH was observed. Other QTLs with highly significant LOD scores were located in chromosomes 2 and 6. The QTL in chromosome 2 (*FIRQV2.1*/*FIRQV2.2*) is mainly involved in FIR, although the other two minor QTLs with lower significance were detected in the same region. The allele of Ved mitigated the climacteric behavior, increasing FIR and diminishing ABS. Using a conservative strategy, we could delimit the QTL in a region of around 28 cM, between physical positions 3,049,874 and 15,771,889 bp (Fig. 4). The QTL in chromosome 6 correlated with chlorophyll degradation, with the Ved allele triggering this symptom. *CDQV6.1* is located in a region of 11 cM, with the maximum LOD score at 7,435,564 bp. Other nine QTLs (in chromosomes 3, 5, 6, 7, 10, and 11) with slight effects in different aspects of climacteric ripening were detected, although their LOD scores were around the threshold of significance. All of them were also detected in some of the individual-block mapping experiments in similar positions and with the same direction of additive effect.

**Table 2:**
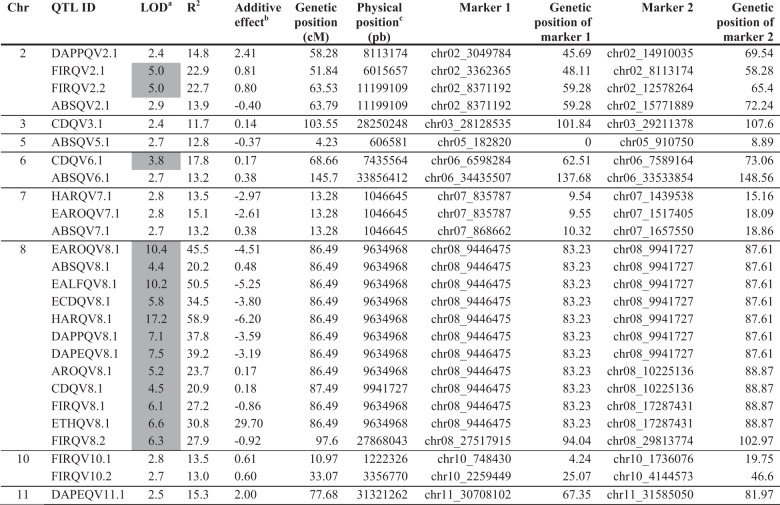
QTLs for climacteric ripening and ethylene production traits detected using the mean of phenotypic values for all the blocks

^a^Presented the QTLs with LOD > 2.4, and QTLs with a value of LOD > 3 are shaded in gray

^b^Sign of the additive effect of the Ved allele

^c^Physical position in version v.3.6.1 of the melon genome

Due to the high effect of *ETHQV8.1* in climacteric ripening, other minor QTLs could be masked and poorly detected in our analysis. To minimize this effect, we separated the RIL population into two subsets, each of them with *ETH8QV8.1* fixed for the Ved or the PS allele (VIII-Ved, *n* = 34 and VIII-PS, *n* = 55). For each subset, we did an interval mapping experiment using LOD > 3 as threshold (Supplementary Table 2B) that revealed the same QTLs but with higher significance and some new potential QTLs. When *ETHQV8.1* is fixed for the Ved allele, all the RILs are climacteric to a greater or lesser degree, so we should increase our power of detection for QTLs that are modulating ethylene production or their physiological responses. The principal factors detected in the previous analysis and in the VIII-Ved subset were in chromosomes 2, 6, and 8 (Fig. 4); other new potential QTLs were located in chromosomes 5 and 9. For the subset VIII-PS, we observed non climacteric and climacteric lines, but in any case the maximum ethylene production surpassed 50 µL kg^−1^ h^−1^, suggesting QTLs that triggered climacteric ripening later or with lower intensity. Furthermore, with this approach we detected the same QTLs in chromosomes 2, 3, 5, and 7, and also a new one at the top of chromosome 8 (Fig. 4).

### Validation and fine mapping of ETHQV8.1

In order to validate *ETHQV8.1*, we used introgression lines (ILs) with the background of one of the parental lines, Ved or PS, and an introgression of the other parent, PS or Ved, in chromosome 8, respectively. These lines belong to a collection of ILs developed by our team (data not shown). Initially, two IL families were used in this study (720 and 414), derived from two BC_3_S_1_ segregating families that, besides the region of *ETHQV8.1*, still contain a few additional introgressions in other chromosomes (Supplementary Table 3A). Two other more advanced generation ILs, PS8.2 and VED8.2, carrying introgressions in chromosome 8 in homozygosity and free of contaminations in other chromosomes were also included in the analysis.

**Fig. 5:**
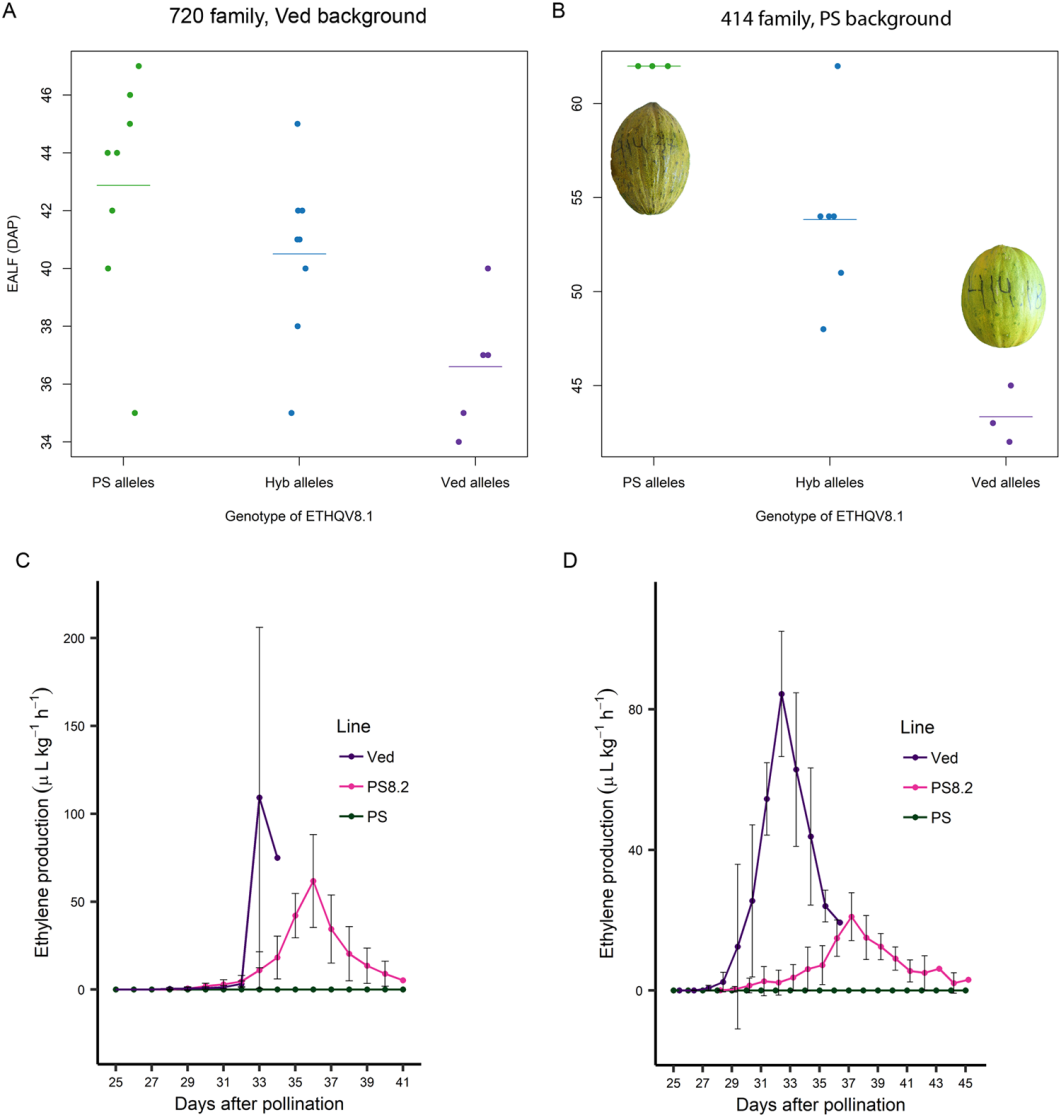
Evaluation of the appearance and the earliness of climacteric symptoms for ILs. **A** In all, 720 family, Ved background, in 2017 summer season. **B** In all, 414 family, PS background, in 2017 summer season, including images to show the external appearance of two individuals of 414 family with different alleles in *ETHQV8.1*. **C** Ethylene production of parental lines and the IL PS8.2 in 2018 summer season (*n* = 4) and **D** in 2019 summer season (*n* = 5), showing the mean of the replicates, and centered in the day of maximum production

We evaluated the appearance and the earliness of climacteric symptoms for 720 and 414 IL families during the 2017 summer season. The 720 family, segregating for the introgression of PS in the genetic background of Ved, presented in all cases climacteric fruits, with presence of aroma, abscission layer, and in most cases change of color (Supplementary Table 3B). However, the earliness of the climacteric symptoms was delayed in the ILs carrying the PS allele of *ETHQV8.1*, either in heterozygosity or homozygosity. The evaluation of the 414 family, containing a Ved introgression in the genetic background of PS, showed clear differences between fruits carrying the Ved allele of *ETHQV8.1*, presenting various degrees of climacteric fruits, in comparison to those carrying the PS allele in homozygosis, which were non-climacteric (Supplementary Table 3B). There is a clear association between the genotype of *ETHQV8.1* and EALF, with the Ved allele decreasing the number of DAP of appearance of the climacteric symptom in both genetic backgrounds (Fig. 5A, B). Statistically, the values were significantly different between Ved and both PS and Hyb (*p*-values of 0.004 and 0.024, respectively) for the 720 family, and between all the possible combinations for the 414 family (*p*-values 0.03 PS-Hyb, 0.007 Ved-Hyb and <0.001 PS-Ved).

To further confirm the role of *ETHQV8.1* in the onset of climacteric ripening, we evaluated the appearance and the earliness of climacteric symptoms for ILs PS8.2 and VED8.2 during the summer season in 2018 and 2019 (Table 3). PS8.2, carrying an introgression of PS in the genetic background of Ved, presented a climacteric behavior during both years, but it resulted delayed when compared to Ved. A significant delay was observed in chlorophyll degradation and abscission layer formation, especially in 2019; and also in the level of abscission, more severe in 2018 (Supplementary Fig. 4). On the other side, VED8.2, carrying a Ved introgression in the genetic background of PS, showed varying degrees of climacteric fruits. The effect was observed both years, but more clearly in 2019, when all the fruits showed a climacteric phenotype, with sweet aroma production and abscission layer formation, while in 2018 only 50% of the fruits presented this climacteric phenotype. Nevertheless, the Ved allele of *ETHQV8.1* alone does not recover the phenotype of the climacteric parent completely (Supplementary Fig. 4), demonstrating the polygenic nature of climacteric fruit ripening and its interaction with environmental factors.

**Table 3:** Basic statistics for climacteric ripening traits in the parental lines and the ILs (PS8.2 and VED8.2) evaluated in 2018 and 2019

		2018				2019			
Trait	Line	Median	Mean	SD	*p*-value	Median	Mean	SD	*p*-value
EARO	VED	33	33.00	0.00	0.235	32	31.40	1.52	0.0659
	PS8.2	33.5	34.25	1.89		34	35.00	3.46	
	PS	54.5	54.5	2.12	0.5	55	55	0	0.000075
	VED8.2	47.5	48.2	11.2		43	44	3.32	
ECD	VED	33	33.25	0.50	0.595	31	31.60	2.19	0.0191
	PS8.2	33.5	33.75	1.71		35	36.00	2.55	
	PS	41	41	9.9	0.836	55	52.6	5.37	0.00116
	VED8.2	36.5	39	10.9		41	40	2	
EALF	VED	33	33.00	0.00	0.000105	32	32.20	2.28	0.0128
	PS8.2	35	35.20	0.50		35.5	37.75	2.75	
	PS	54.5	54.5	2.12	0.746	55	55	0	0.0003
	VED8.2	54	55.5	3.7		47	46	3.32	
HAR	VED	34	33.80	0.50	0.000115	36	36.40	1.67	0.000476
	PS8.2	40	39.75	1.26		43	43.00	2.00	
	PS	54.5	54.5	2.12	0.749	55	55	0	0.0207
	VED8.2	54	55.5	3.7		50	50.4	3.58	
ABS	VED	3	3	0	0.000423	1	1.4	0.894	0.217
	PS8.2	1	1.25	0.5		1	0.8	0.447	
	PS	0	0	0	1	0	0	0	0.0133
	VED8.2	0	0	0		1	1	0.707	
FIR	VED	3.4	3.28	0.314	0.0114	3.74	3.51	0.803	0.9
	PS8.2	4.29	4.26	0.446		3.22	3.44	0.837	
	PS	4.38	4.38	0.177	0.0204	3.4	3.11	0.885	0.012
	VED8.2	2.15	1.95	0.863		1.69	1.77	0.27	

In addition, we measured the ethylene production in both parental lines and the IL PS8.2. We could validate the effect of *ETHQV8.1* in DAPE, DAPP, and ETH, observing a weaker climacteric phenotype compared to Ved (Fig. 5C, D). In 2018, due to the extreme abscission phenotype, we could not clearly observe the peak of ethylene production in Ved, but we could detect a significant delay of the ethylene peak in PS8.2, compared to Ved (*p*-value of 0.007) (Fig. 5C). In 2019, we could clearly observe the peak of ethylene production in all fruits (Fig. 5D). Statistically, the differences were significant for the three measured phenotypes DAPE, DAPP and ETH (*p*-values of 0.047, 0.0003, and <0.001, respectively).

We increased the SNP density between the flanking markers of the *ETHQV8.1* interval (Supplementary Table 3A, flanking markers shaded in red). The most informative segregating ILs were genotyped, and three of them had recombinations within the QTL interval (720.17, 414.1, and 414.17) (Supplementary Table 3C). Progeny seed of 414.1 and 414.17 were germinated, and a subset of ILs were evaluated during the 2017 autumn season. None of the 11 plants of the 414.1 family showed abscission, although some fruits presented a subtle abscission layer around 55 DAP or later (Supplementary Table 3D). In contrast, nine from the 15 plants from the 414.17 family presented abscission between 44 and 52 DAP. The family 414.1 was segregating for a small introgression in chromosome 8, which partially included *ETHQV8.1*, from the initial position 9,446,475 to 9,603,217 bp (Supplementary Fig. 5); the phenotype of this family ruled out that *ETHQV8.1* was located in this interval, since the subtle abscission layer observed was not associated with the genotype of the lines. The family 414.17 segregated in the upstream part of *ETHQV8.1*, from 9,446,475 to 9,757,323, and both the earliness and the intensity of abscission were strongly associated with the genotype of the line in the segregating part of the introgression (Supplementary Fig. 5). Both families delimit *ETHQV8.1* to a region of 154.1 Kb, which contains the maximum LOD peak obtained in the QTL mapping (Supplementary Table 3E).

### GWAS for climacteric ripening in a collection of melon accessions

We performed an association study for aroma production (assessed subjectively by smelling the fruit) in a subset of 211 melon accessions corresponding to the ssp. *melo* (Supplementary Table 4)^30^. We identified two close association signals in the top of chromosome 8 (−log10[P] = 7.0 and (−log10[*P*] = 6.0, respectively; Supplementary Fig. 6). One of the signals coincides with the *ETHQV8.1* region of 154.1 Kb identified by genetic mapping.

## Discussion

### A gradient of climacteric ripening was observed in the Ved × PS RIL population

The main approach used to determine the genetic control of climacteric fruit ripening has been the characterization of tomato ripening mutants in which the climacteric wild type is compared with the non-climacteric mutant^5^. As an essential part of phenotyping of ripening mutants, ethylene production is evaluated during the ripening process. Generally, the hormone is measured in detached fruits, which are collected when they have acquired the competence to ripen but before the ripening process begins. Although this strategy has been widely used and with demonstrated efficiency, it presents some disadvantages when applied to mapping populations, as it requires enough space to store containers to contain the fruits during the entire ripening period. Previous knowledge of the ripening date of each line is needed to adjust the harvest point. In addition, as the in planta development is interrupted, it may lead to potential alterations in ripening. The quantitative, non-invasive method we used to monitor the ethylene peak allowed us the observation of ethylene responses and the timing of their appearance, and their intensity in planta^31^. The comprehensive evaluation of climacteric ripening in the Ved × PS RIL population has led to a complex and diverse group of phenotypes. From the two extreme phenotypes observed in the parental lines, the segregating RIL population displayed several combinations differing not only in quantity and earliness of ethylene production, but also in ripening-associated phenotypes. Some ripening-associated phenotypes were not present in highly climacteric lines, e.g. RIL 124 presented high production of ethylene, very sweet aroma, and abscission in all blocks, but it did not change external color; whereas RIL 213 showed medium levels of ethylene, aroma, and a striking change of color to bright yellow without abscission layer formation.

Some RILs that produce as little as 5% of the ethylene produced by Ved are phenotypically very similar, indicating that only a minimal threshold of the hormone (around 2 µL kg^−1^ h^−1^) is necessary to trigger climacteric ripening. However, some common characteristics were observed in RILs producing a low quantity of ethylene: they tended to ripen later, the ethylene peak was less defined in comparison with lines that produced higher levels of ethylene, and their phenotype was dependent on the environment, showing wider variation depending on the block. We hypothesize that the amount of ethylene produced by these RILs is very close to the minimum threshold necessary to trigger the climacteric behavior, so any environmental alteration that reduces it may have a critical effect resulting in a non-climacteric fruit. Depending on the stability of the observed climacteric phenotypes among the RILs, we could identify three different ripening patterns: climacteric lines, which had at least one symptom of climacteric ripening in all blocks; unstable climacteric lines, showing an inconsistent behavior in different blocks; and non-climacteric lines in all blocks. In general, unstable climacteric lines developed melons clearly climacteric in some blocks, with detectable ethylene, aroma, and abscission layer formation, but totally non-climacteric in other blocks. A PCA accurately groups the RILs according with this classification and evidences the high proportion of climacteric and unstable climacteric lines in comparison with non-climacteric ones in this RIL population (Supplementary Fig. 7).

The phenotypic distribution anticipates the existence of multiple QTLs with variable effects modulating climacteric ripening. A panel of melon accessions was previously classified in degrees of climacteric ripening, using principally FIR and abscission (ABS) as indicative traits^32^. Although their phenotyping was not as extensive as in our work, they suggested a non-absolute classification of melon varieties according to this trait. These complex and polygenic nature of the trait was also demonstrated by previous works that characterized several QTLs for climacteric ripening or their associated effects. A RIL population obtained from the cross between Ved and PI 161375, a non-climacteric accession, was used to identify two genes responsible for abscission layer formation, *Al-3* and *Al-4* in chromosomes 8 and 9, respectively, and four QTLs affecting the amount of ethylene production in chromosomes 1, 2, 3, and 11^25^. An IL population founded by the same exotic parental, PI 161375, and PS, both non-climacteric, led to the identification of QTLs for climacteric ripening (*eth3.5*) and flesh firmness (*ff2.2*, *ff3.5*, *ff8.2*, *ff8.4* and *ff10.2*)^33^. Another study implicating the same PI 161375 x PS population revealed a second QTL (*ETHQV6.3*) that also rescued the climacteric phenotype in the PS background when the exotic allele was present^26^. The presence of the exotic allele in either *eth3.5* or *ETHQV6.3* promoted climacteric ripening, but the combination of both conferred an earlier and more intense climacteric phenotype^26^. One QTL in chromosome 10 and possibly a second one in chromosome 7 were identified, using an IL population with the background of Ved and introgressions from the non-climacteric Japanese “Ginsen makuwa” cultivar from the ssp. *agrestis*^27^. The present work has characterized several QTLs interfering with different aspects of climacteric ripening in chromosomes 2, 3, 5, 6, 7, 8, 9, 10, and 11, using a combination of QTL mapping experiments. Several of these QTLs could be allelic to the QTLs previously described (Supplementary Table 5), since they co-localize in the same physical positions. The minor effect of most of the described QTLs fits with the hypothesis of a complex trait with polygenic inheritance, displaying a continuum range of climacteric intensity. In this work, the use of two phylogenetically close varieties as Ved and PS, both commercial and belonging to ssp. *melo*, allowed to identify several QTLs involved in climacteric ripening.

### A major QTL, ETHQV8.1, is sufficient to trigger climacteric ripening

Among the identified QTLs, *ETHQV8.1* was most significant in affecting both ethylene production and ripening-associated phenotypes. For all the recorded traits except WEP, a highly significant QTL was mapped in an almost identical interval as *ETHQV8.1*, around the physical position 9,634,968 bp of chromosome 8. In all of these traits the Ved allele is causing a stronger climacteric phenotype, explaining between 20.2% and 58.9% of the variance. Furthermore, in the RIL subset Ved-VIII, which contains only the RILs with the Ved allele in the *ETHQV8.1* interval, none of them is non-climacteric. It is possible that *ETHQV8.1* is allelic to *Al-3*, a major gene described in the PI 161375 x Ved RIL population as essential to trigger fruit abscission and endogenous synthesis of ethylene^25^. However, the physical position of *Al-3* could not be determined as the genetic map where it was described was exclusively based on AFLP markers, precluding the comparison of the QTL positions.

In order to validate *ETHQV8.1*, we generated ILs with the PS and Ved background and with introgressions containing PS or Ved alleles in the *ETHQV8.1* interval, respectively. The evaluation of two ILs, VED8.2 and PS8.2, with PS and Ved background, respectively, proved the contribution of *ETHQV8.1* to the climacteric phenotype. According to the polygenic control of the trait, the IL with climacteric background (PS8.2) did not lose completely the climacteric phenotype. However, a significant delay was observed in climacteric symptoms caused by a delay and also a decrease of the ethylene production. This effect is different to the one reported using mutants of the *CmNAC-NOR* gene with in the “Charentais Mono” background (*cantaloupensis* group), where the mutants showed a delay in ethylene production and subsequently, in climacteric symptoms appearance, but without a reduction in the amount of ethylene^28^. On the other side, the IL with non-climacteric background (VED8.2) presented a smooth climacteric phenotype, but highly dependent on the environment.

To fine map *ETHQV8.1*, we selected two individuals that had recombined inside the QTL interval and performed an evaluation of their progenies in autumn 2017. The climacteric behavior was less intense to the one observed during summer, as expected, since even below controlled conditions, fruit ripening is altered. The climacteric fruits did not change substantially their external color and did not produce aroma, nevertheless, they produced abscission layer and fell from the plant, suggesting that in the PS background, fruit abscission is less environment-dependent. Therefore, the phenotyping of two progenies of recombinant individuals from the family 414 reduced *ETHQV8.1* to an interval between the positions 9,564,672–9,757,323 bp. Using the version v4.0 of the annotation of the melon genome^34^, 14 candidate genes are located inside the interval (Supplementary Table 6). Tentatively, three of them could be discarded because they are not expressed in fruit tissues in Melonet-DB^35^; by functional annotation, three genes could be related to *ETHQV8.1*, encoding an ethylene-responsive transcription factor *ERF024* (MELO3C024520), a serine/threonine kinase CTR1-like (MELO3C024518) and a protein *ROS1* (MELO3C024516). MELO3C024518 has six non-synonymous variants between PS and Ved. *CTR1* is a negative regulator of ripening that interacts physically with ethylene receptors^36^. In the absence of ethylene, the ethylene receptor activates *CTR1*, which leads to the inhibition of the downstream transduction pathway; when ethylene is present, the ethylene receptor terminates the activation of *CTR1*, thus releasing ethylene responses^37^. MELO3C024516 has 10 non-synonymous variants between Ved and PS. *ROS1* encodes a demethylase, and demethylation has been reported as one of the main mechanism regulating fruit ripening^15,38^. In tomato, the *ROS1* orthologue *DML2* is a demethylase governing fruit ripening^14^. MELO3C024520 has not sequence variants between PS and Ved causing changes in the protein, and its expression in fruit is low^34^.

**Fig. 6:**
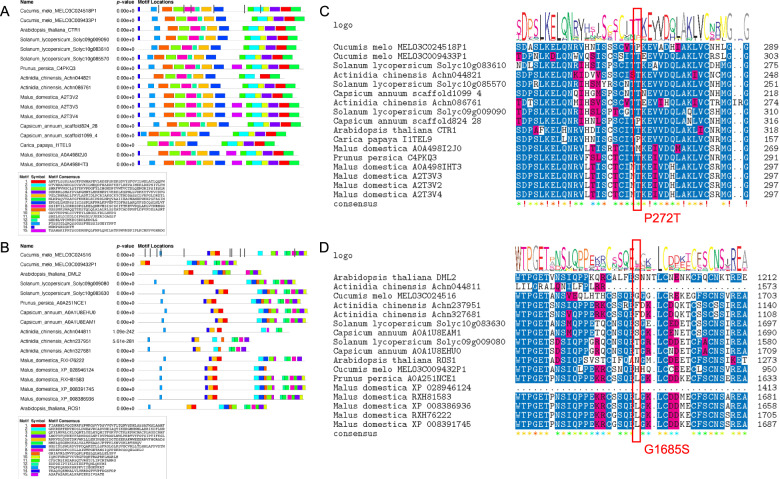
Functional analysis of the two candidate genes of *ETHQV8.1*. **A**, **B** identification of conserved protein motifs using orthologs from several species (*Arabidopsis*, tomato, peach, kiwi, apple, papaya and pepper); the gray bars represent non-synonymous SNPs between PS and Ved. **C**, **D** Multiple protein alignment containing the most likely causal variant for each gene; in red box and letters, the aminoacid change in melon proteins between PS (before the position) and Ved (after the position)

To further investigate the relevance of the non-synonymous variants between Ved and PS within the two most likely candidate genes MELO3C024516 and MELO3C024518, we obtained a phylogenetic tree of the homologous proteins in other climacteric (tomato, peach, kiwi, apple, papaya) and non-climacteric (pepper) species, also including *Arabidopsis* (Supplementary Fig. 8). We identified the closest orthologs to MELO3C024516 (*CmROS1*) and MELO3C024518 (*CmCTR1*) using a phylogenetic analysis and selected them to study the conserved motifs containted in these proteins (Fig. 6A, B). Both proteins were highly conserved among species and 15 significant motifs were identified in each of them. *CmCTR1* and *CmROS1* proteins were complete and included all the motifs observed in the tomato and *Arabidopsis* orthologs. Of the six non-synonymous SNPs contained in *CmCTR1* (Fig. 6A), three were located within conserved motifs, potentially affecting the protein. Among them, two were conservative aminoacid replacements (E386Q and E628D), which most likely might not impact protein function, and one (P272T) was contained in motif 8 and was non-conservative (Fig. 6C). Ved, the climacteric parent, presented a threonine at this position, as well as most analyzed climacteric species (tomato, kiwi, apple, peach); meanwhile PS presented a proline, as one of the two orthologs of the non-climacteric pepper. However, the papaya ortholog also had a proline in this position. For *CmROS1*, we observed 10 non-synonymous changes and two of them were contained within conserved protein motifs (Fig. 6B). The first one, contained in motif 13, was a non-conservative change (E250G), although the aminoacid was quite variable in this position among species (20% R, 20% V, 53% I, and 6.7%E). The second one, located in motif 8, was from glycine (aliphatic) in PS to serine (polar) in Ved at position 1685 (Fig. 6D), but again this position was not highly conserved. According to the non-synonymous variants observed within the parents of this population for the two candidate genes, their expression levels in the melon expression atlas^35^ and the previous knowledge about their function in other climacteric species, both candidate genes could be involved in climacteric ripening and be the underlying gene for *ETHQV8.1*. Further experiments, such as the generation of knock-out mutants and the analysis of expression levels during fruit ripening are needed to validate either of them as the underlying gene for *ETHQV8.1*.

In addition, we performed a GWAS analysis for production of aroma in a subset of 211 re-sequenced accessions of the *melo* ssp. Two close association signals were detected in chromosome 8, one of them located in the 154.1 Kb that contains the above-mentioned candidate genes (Supplementary Table 7). This result suggests that *ETHQV8.1* may be important for regulating climacteric fruit ripening in melon germplasm.

### Other minor QTLs interact to modulate the climacteric response

In addition to *ETHQV8.1*, other genetic factors are involved in the ripening process in our RIL population. In general, they are not affecting the amount of produced ethylene, but other traits as flesh firmness or external fruit color. Although some of them, as *FIRQV2.1*/*FIRQV2.2* and *CDQV6.1*, presented a highly significant LOD (>3.5), in most cases they have a limited effect in ripening from a global perspective, in contrast to *ETHQV8.1*.

Two regions, in chromosomes 2 and 7, seemed to be implicated in fruit ripening in a general way, since QTLs for different traits were detected. In chromosome 2, we identified two QTLs for FIR, which colocalize with other QTLs for DAPP and ABS. Furthermore, the other two QTLs for EARO and HAR are detected in the Ved-VIII subset and a forth one for FIR in the PS-VIII. In all cases, the Ved allele was diminishing or delaying the climacteric response (Table 2 and Supplementary Table 2B). We hypothesized that a unique QTL affecting the earliness of ethylene production is located between the physical positions 3,049,784–15,771,889 bp of chromosome 2, which contains 669 annotated genes. We did not identify QTLs for the qualitative traits (ARO, CD) even in the PS-VIII subset, suggesting that *FIRQV2.1*/*FIRQV2.2* is incapable to trigger the autocatalytic ethylene production by itself. Previous QTLs in chromosome 2 have been described for FIR and ETH (Supplementary Table 5). The second region was identified in chromosome 7, presenting three overlapping QTLs for HAR, EARO, and ABS, with a maximum LOD of 2.8 in the physical position 1,046,645 bp, contributing positively to climacteric ripening and explaining around 15% of the variance (Table 2). A QTL in a similar position was mapped using the PS-VIII subset for HAR, with a LOD of 3.45, so possibly this QTL is one of the factors that contribute to trigger climacteric ripening when *ETHQV8.1* is fixed for PS alleles. The CI for the QTL is delimitated by the physical positions 835,787–1,657,550 bp and contains 129 genes. Although a QTL for climacteric ripening in chromosome 7 was not described before, an IL with an exotic introgression in this region that was much less climacteric than the control has been reported^27^; however, an additional introgression in chromosome 10 that actually modified climacteric ripening made it difficult to assess the contribution of chromosome 7.

*CDQV6.1* controls one process exclusively, the chlorophyll degradation that leads to a visible change of color. It was detected with significant LOD in the mapping experiments with three blocks (Supplementary Fig. 3C) and with Ved-VIII subset (Supplementary Table 2B), in addition to the one with mean values (Table 2). The Ved allele increased CD in a climacteric background, as the QTL was significant in the Ved-VIII subset, but we could not detect it in the PS-VIII subset. Mutants with a similar phenotype had been studied in tomato and pepper, identifying a STAY-GREEN protein as responsible for their inability to degrade chlorophyll at the onset of ripening^39^. *CDQV6.1* is located between the physical positions 6,598,284–7,589,164 bp of chromosome 6 and contains 106 genes. The interval did not contain *CmNAC-NOR* (coordinates 27,663,292–27,665,351 bp of chromosome 6), in fact no LOD peak was detected at these positions; probably the interactions between the QTL with the genetic background and the environment caused the loss of detection for the effect of this gene in our population.

QTLs for flesh firmness were mapped in chromosomes 10 and 8. Two QTLs, *FIRQV10.1* and *FIRQV10.2*, were located in chromosome 10 (Table 2). Since both are increasing FIR when the Ved allele is present and are close from each other, probably there is a single QTL in the interval 748,430–1,736,076 bp (Supplementary Fig. 3E). This interval contains 154 genes. *FIRQV8.2* was mapped in chromosome 8, around 10 Mb downstream *ETHQV8.1*, with a maximum LOD of 6.3 in the position 27,868,043 bp (Table 2). A QTL for FIR in similar positions was detected using the Ved-VIII subset, with a maximum LOD = 3.4 (Supplementary Table 2B). Fruit texture has been broadly studied in melon due to its great importance from the commercial perspective: the market desires climacteric varieties that remain firm during the postharvest stage^22,27,40^. Unlike in tomato, the softening of fruit flesh in melon is partially controlled by ethylene, so the non-climacteric types also ripen entering in a phase of sugar accumulation and decrease of flesh firmness^24^. A comparative transcriptomic analysis of climacteric and non-climacteric varieties showed that the set of enzymes modifying cell-wall metabolism was different in Ved (polygalacturonases, glucan endo-1,3-β-glucosidases, and β-d-xylosidases) and PS (fascilin-like arabinogalactanan protein)^22^.

The mapping experiment that discriminates the RIL population depending on the haplotype for *ETHQV8.1* allowed us to propose three new QTLs that were masked in the main QTL mapping analysis and to support some of the minor QTLs already detected (Fig. 4), in addition to gaining some insight about how these genetic factors work. Using the Ved-VIII subset, we could detect two QTLs related to ethylene production, *WEPQW5.1* and *ETHQW9.1* (Supplementary Table 2B). *WEPQW5.1* could modulate the rate at which the ethylene production increases during the onset of ripening, until reaching the peak; so it could be participating in promoting the autocatalytic synthesis of the hormone, rather than interacting with the physiological responses. *ETHQW9.1* may be regulating the amount of ethylene produced but may not have the ability of initiating the production itself, which would explain why we could not map it using data from the whole population. *ETHQW9.1* could be allelic to a second major gene implicated in fruit abscission in the distal part of chromosome 9 (Supplementary Table 5). On the opposite side, the PS-VIII subset suggested the existence of minor QTLs in chromosomes 3, 5, 7, and 8 that could be provoking a slight climacteric ripening. Most of them were already mapped in very similar positions (Supplementary Fig. 3 and Fig. 4) except *EALFQX8.3* and *DAPPQX8.3*. The Ved allele for these QTLs were accelerating the climacteric response, but they were not detected for the qualitative traits (ARO, CD) and were therefore probably not sufficient to trigger ethylene production.

The ripening behavior of the RIL population obtained by crossing a highly climacteric and a non-climacteric melon type suggests that climacteric fruit ripening is a complex trait in melon. A major QTL *ETHQV8.1* is sufficient to trigger climacteric ripening, although additional minor QTL are important to modulate the climacteric response. The genomic interval containing *ETHQV8.1*, which was also identified in a parallel GWAS experiment, contains at least three candidate genes that may be related to fruit ripening. Additional experiments are needed in order to identify the gene responsible for the *ETHQV8.1* climacteric phenotype. This work allowed the identification of a new component that contributes to this complex trait, which may be useful in breeding programs aimed at obtaining long-shelf life melon types.

## Materials and methods

### Plant material

Plant material was grown in a greenhouse at Caldes de Montbui (Barcelona). Plants were pruned weekly and pollinations were executed manually, limiting development to only one fruit per plant. The harvest point was determined by the following criteria: (a) abscission date when the fruit abscised; (b) after seven days of the appearance of any symptom of climacteric ripening in absence of abscission; (c) at 55–62 days after pollination (DAP) when fruits were non-climacteric.

The RIL population used in this work was described in Pereira et al. Briefly, the population was developed from a cross between Ved (ssp. *melo*, *cantalupensis* group) and PS (ssp. *melo*, *inodorus* group) and contained 89 RILs. Five blocks (T1-T5), consisting in a unique fruit per RIL and 1–3 fruits per control line (Ved, PS and the F1 (Hyb)), were grown during the summers of 2015 and 2016^29^. The three blocks from 2015 (T1-T3) were sowed and planted with a delay between them of 20 days approximately. Although all the phenotypic evaluations were performed within the summer period (July-September) and the conditions in the greenhouse were partially controlled, we considered them as independent trials. The two blocks from 2016 (T4-T5) were planted at the same time, but were treated as independent trials to have a homogeneous design.

Two introgression line (IL) collections were obtained from the F1 of the Ved x PS cross after three backcrosses with the recurrent parentals Ved and PS, respectively (data not shown). Two families of ILs (720 and 414), containing introgressions in chromosome 8 of PS and Ved in the background of Ved and PS, respectively, were evaluated during the summer of 2016 (family 720, *n* = 18 and family 414, *n* = 12, respectively) and the progenies of two recombinant individuals of the 414 family (414.1, *n* = 11 and 414.17, *n* = 15) during the autumn of 2017. Two advanced ILs (PS8.2 and VED8.2), containing introgressions in chromosome 8 of PS in the background of Ved, and Ved in the background of PS, were evaluated during the summer of 2018 (*n* = 4) and 2019 (*n* = 5).

Melon accessions for genome-wide associacion studies (GWAS) were obtained from the National Mid-term Genebank for Watermelon and Melon (Zhengzhou, China), the Zhengzhou Fruit Research Institute (Chinese Academy of Agricultural Sciences) and the US Department of Agriculture. Plants were grown in Zhengzhou (Henan province), Sanya (Hainan province) and Changji (Xinjiang province) in 2015 and 2016 and were previously described^30^. Because of poor adaptation, some accessions were evaluated in one or two locations, and three replicates were evaluated at each location.

### Phenotyping of climacteric ripening traits

Ripening-related traits were evaluated as qualitative (production of aroma (ARO), chlorophyll degradation (CD), and abscission (ABS)) or quantitative (rest of traits) (Table 1). Traits were divided in four different groups according to the physiological response to the production of ethylene: biosynthesis of volatiles leading to a sweet aroma (ARO and EARO), change of color mainly due to chlorophyll degradation (CD and ECD), abscission layer formation in the pedicel of the fruit, provoking abscission in some cases (EALF, ABS and HAR) and softening of fruit flesh (FIR). The visual inspection of melon fruits, attached to the plant, was performed daily, from approximately 25 DAP until harvest. In addition, individual pictures of the fruits were obtained weekly. ARO and CD were recorded as 0 = absence and 1 = presence. ABS was recorded using an index from 0, no abscission layer formation (ALF); 1, subtle and/or partial ALF; 2, almost complete ALF with obvious scar; and 3, total ALF generally with fruit abscission. The aroma production was evaluated each day by smelling the fruits. The firmness of fruit flesh was measured at harvest using a penetrometer (Fruit Test^TM^, Wagner Instruments), in at least three regions of the fruit (distal, proximal and median), and the mean value was registered. Ripening-related traits were phenotyped in the five blocks of the RIL population (T1-T5). ILs were evaluated in 2017, 2018, and 2019 following the same criteria.

For GWAS analysis, climacteric ripening was recorded evaluating aroma production by smelling the fruits. Accessions without aroma were recorded as “0” and accessions with strong aroma were recorded as “2“.

### Ethylene production

Ethylene production in attached melon fruits was measured using gas chromatography – mass spectrometry (GC-MS) as previously described^31^. Two blocks (T3 and T4) of RILs were evaluated for this trait. Ethylene production in attached fruits was measured in 66 RILs both years, in 17 RILs only 1 year and six RILs were excluded from the analysis due to infections or a wrong fruit set. In addition, IL PS8.2 and both parental lines were evaluated during the 2018 and 2019 summer seasons.

The ethylene peak was monitored during the ripening period customizing the design for each RIL. The first measurement before the onset of ripening was determined for each RIL based on previous evaluations, due to the segregation of earliness of ethylene production. For climacteric lines, the atmosphere of the chamber containing the fruit was measured every other day while ethylene was undetectable and every day after ethylene detection; the measurements were stopped when: a) fruit abscised from the plant or b) ethylene production decreased during at least two consecutive days. For non-climacteric lines, the atmosphere of the chamber was examined at least every three days, confirming that they did not produce any detectable amount of ethylene during fruit ripening.

For the ILs evaluated in 2018 and 2019 (PS8.2 and VED8.2), the measurements were started at 25 DAP, and stopped when: (a) fruit abscised from the plant or (b) 5 days after the formation of the abscission layer. The non-climacteric parent PS was examined using the same criteria as in the RILs.

To better characterize the ethylene peak, four traits were defined: maximum production of ethylene in the peak (ETH), earliness of ethylene production, representing the first day when ethylene was detectable (DAPE), earliness of the ethylene peak (DAPP), and width of ethylene peak (WEP), the latter calculated subtracting DAPP and DAPE (Table 1). For non-climacteric lines, the earliness of the trait was considered as missing data.

### DNA extraction and genotyping

DNA extractions were performed from young leaves following the CTAB protocol^41^ with some modifications^29^. The IL families 720 and 414 and the ILs PS8.2 and VED8.2 were genotyped with SNPs using the KASPar SNP Genotyping System (KBiosciences, Herts, UK). KASPar assay primers were designed following the protocol of LGC Genomics (Supplementary Table 8A). The genotyping of SNPs across the genome was performed using the high-throughput genotyping system Biomark HD, based on Fluidigm technology, with 96 × 96 chips. Additional SNPs located within the flanking SNPs of *ETHQV8.1* were genotyped by qPCR (Supplementary Table 8B). All the SNPs used were obtained in silico from the re-sequencing of both parental lines^42^.

The re-sequenced accessions used in GWAS are described in Zhao et al.^30^.

### Genetic map construction and QTL mapping

The genetic map, containing SNPs and INDELs genotyped by genotyping-by-sequencing (GBS), was previously described in^29^. Phenotypic data was used to perform a QTL mapping analysis^43^ with the interval mapping procedure in each block individually, T1-T5 for ripening-related traits and T3-T4 for ethylene traits. The threshold of significance was fixed at LOD > 2.5^44^ and the 1-LOD confidence interval (CI) was used to locate the QTL. QTLs were considered significant only when at least three of them were detected in similar positions and contributed to ripening in the same direction. Chromosomes that did not contain any significant QTL were not represented.

In order to increase the statistical power, a second interval mapping analysis was performed using the mean values for each RIL (LOD threshold = 2.4). A Principal Component Analysis (PCA) based on a subset of RILs and variables, evaluated in five blocks and without missing data, showed that, when extracting the effect of the line, the environmental effect was not associated with the year (Supplementary Fig. 9).

To name the QTLs, the first letter corresponds to the trait code (Table 1), followed by a “Q”, a letter identifying the experiment (“V”, “W”, and “X” in the mapping with the mean values, the subset VIII-VED and the subset VIII-PS, respectively), a number indicating the chromosome, a dot and a digit to differentiate QTLs in the same chromosome^45^.

### Phylogenetic analysis and multiple alignments

To obtain the orthologs of the candidate genes, the protein sequences from melon candidate genes were blasted in Plaza^46^, Uniprot and NCBI. The most similar proteins from climacteric (tomato, peach, kiwi, apple, papaya) and non-climacteric (pepper) species, also including *Arabidopsis*, were downloaded and used to generate a phylogenetic tree in NGphylogeny.fr^47^. The algoritm MAFFT was used for the alignment^48^, BMGE for the alignment curation^49^, and PhyML+SMS for the tree inference^50^. Afterwards, the sequences belonging to the same clade as the melon candidate gene were used to identify the conserved protein motifs using MEME (MEME Suite 5.1.1)^51^, allowing 15 motifs at maximum. Later on we generated a protein multiple alignment in R (package msa^52^) with the function msaMuscle.

### Statistical analyses

All the statistical analyses and graphical representations were obtained using the software R v3.2.3^53^ with the RStudio v1.0.143 interface^54^.

The PCA was performed using R package “factoextra”. To obtain the correlation matrix among traits we calculated the Pearson coefficient with the R package “Hmisc” and the visualization of data was performed with “corrplot”. To compute these functions the data should not contain any missing data. In order to include in the analysis the non-climacteric lines without any phenotypic symptoms, EARO, ECD, EALF, DAPP and DAPE values were imputed with the latest harvest date value (55–62 DAP), and for WEP as the maximum value of this variable (8 days). The lines without any data for ethylene production were excluded of the PCA and correlation analysis.

Mean comparisons between the IL groups were performed with the function “pairwise.t.test” from the package “stats”. To represent the EALF in the non-climacteric lines, we substituted the missing value by 55–62 DAP, as explained above.

### Genome-wide association study

Considering the different independent domestication events in melon, we only used *C. melo* L. ssp. *melo* accessions described in ref. ^30^. Only SNPs with minor allele frequency ≥ 0.05 and missing rate ≤ 0.4 were used to carry out GWAS. This resulted in 1,923,713 SNPs that were used in GWAS. We performed GWAS using the Efficient Mixed-Model Association eXpedited (EMMAX) program^55^. Population stratification and hidden relatedness were modeled with a kinship (K) matrix in the emmax-kin-intel package of EMMAX. The *P*-value thresholds for significance were approximately 2.51 × 10^−6^.

## Supplementary information

Supplementary Tables 1, 5 and 6

Supplementary Figure 1

Supplementary Figure 5

Supplementary Figure 7

Supplementary Figure 9

Supplementary Figure 3

Supplementary Figure 8

Supplementary Figure 2

Supplementary Figure 6

Supplementary Figure 4

Supplementary Table 2

Supplementary Table 3

Supplementary Table 4

Supplementary Table 7

Supplementary Table 8
