# Supplementary material for: Genetic dissection of climacteric fruit ripening in a melon population segregating for ripening behavior
Source: Hortic Res. 2020 Nov 1;7:187. doi: 10.1038/s41438-020-00411-z (PMC7603510; doi:10.1038/s41438-020-00411-z)
Supplement: Supplementary file 1 — Supplementary Tables 1, 5 and 6 [file 41438_2020_411_MOESM1_ESM.docx]

Supplementary Table 1. Basic statistics for climacteric ripening traits in the parental lines.

| **Trait** | **Line** | **Median** | **Mean** | **SD** |
| --- | --- | --- | --- | --- |
| ARO | Ved | 1.00 | 1.00 | 0.00 |
|  | Hyb | 1.00 | 1.00 | 0.00 |
|  | PS | 0.00 | 0.00 | 0.00 |
| EARO | Ved | 34.00 | 34.67 | 2.27 |
|  | Hyb | 34.00 | 35.22 | 3.03 |
|  | PS | - | - | - |
| CD | Ved | 1.00 | 0.83 | 0.39 |
|  | Hyb | 1.00 | 0.78 | 0.44 |
|  | PS | 0.00 | 0.00 | 0.00 |
| ABS | Ved | 3.00 | 2.92 | 0.29 |
|  | Hyb | 3.00 | 2.67 | 0.71 |
|  | PS | 0.00 | 0.00 | 0.00 |
| EALF | Ved | 34.00 | 34.67 | 2.27 |
|  | Hyb | 36.00 | 37.22 | 4.02 |
|  | PS | - | - | - |
| ECD | Ved | 34.00 | 34.80 | 2.30 |
|  | Hyb | 35.00 | 35.86 | 1.95 |
|  | PS | - | - | - |
| FIR | Ved | 2.40 | 2.73 | 1.74 |
|  | Hyb | 2.00 | 1.71 | 1.32 |
|  | PS | 2.45 | 3.17 | 2.14 |
| HAR | Ved | 36.50 | 38.67 | 5.07 |
|  | Hyb | 38.00 | 40.22 | 5.47 |
|  | PS | 61.50 | 59.50 | 5.24 |

Supplementary Table 5. Summary of QTLs described in other studies that map in similar intervals to those detected in our work.

| **Chr** | **Trait** | **QTL** | **Plant material** | **Physical position^1^ (pb)** | **Linked marker** | **References** |
| --- | --- | --- | --- | --- | --- | --- |
| 1 | ETH | *eth2.1* | Ved x PI 161375 | - | E39/M42_20 | ^25^ |
| 2 | FIR | *ff2.2* | PS x PI 161375 | - | CMGA36a | ^32^ |
| 3 | ETH | *eth3.1* | Ved x PI 161375 | - | E43/M44_20 | ^25^ |
| 3 | ETH | *eth3.5* | PS x PI 161375 | 26,669,705 | A_16-C12 | ^26^ |
| 8 | ABS | *Al-3* | Ved x PI 161375 | - | H33/M43_21 | ^25^ |
| 8 | FIR | *ff8.2* | PS x PI 161375 | 5,577,470 | CMTC13 | ^32^ |
| 8 | FIR | *ff8.4* | PS x PI 161375 | 34,764,449 | CMTCN56 | ^32^ |
| 9 | ABS | *Al-4* | Ved x PI 161375 | - | H36/M37_11a | ^25^ |
| 10 | FIR | *ff10.2* | PS x PI 161375 | - | All LG | ^32^ |
| 10 | ABS | *al.10* | Ved x Makuwa | 1,722,720 | CMPSNP528 | ^27^ |
| 11 | FIR | *-* | Collection of accessions | 29,559,098 | PSI_41-B07 | ^31^ |
| 11 | ETH | *eth11.1* | Ved x PI 161375 | - | E35/M35_8 | ^25^ |

^1^When possible, an approximate physical position in the v3.6.1 of the melon genome is presented

Supplementary Table 6. List of potential candidate genes for *ETHQV8.1*.

| **Gene** | **Initial position** | **Final position** | **Description** | **Variants causing changes in the protein** | **Gene expression^1^** | | | | |
| --- | --- | --- | --- | --- | --- | --- | --- | --- | --- |
|  |  |  |  |  | **Fruit flesh DAP22** | **Fruit flesh DAP29** | **Fruit flesh DAP36** | **Fruit flesh DAP43** | **Fruit flesh DAP50** |
| MELO3C024522 | 9603252 | 9605249 | BnaAnng07340D protein | 1 | 12.5 | 16.6 | 19.2 | 14.8 | 11.0 |
| MELO3C024521 | 9623758 | 9626574 | Histone-lysine N-methyltransferase SETD1B-like protein | 2 | 0.0 | 0.0 | 0.0 | 0.0 | 0.1 |
| MELO3C024520 | 9630393 | 9630993 | ethylene-responsive transcription factor ERF024 | 0 | 0.0 | 0.6 | 1.1 | 3.3 | 10.0 |
| MELO3C024519 | 9634048 | 9636531 | Fructose-bisphosphate aldolase | 0 | 125.4 | 71.5 | 103.4 | 93.7 | 72.9 |
| MELO3C024518 | 9638990 | 9648937 | serine/threonine-protein kinase CTR1-like | 6 | 17.6 | 25.1 | 20.6 | 18.0 | 0.7 |
| MELO3C024516 | 9653281 | 9675711 | protein ROS1 | 10 | 3.0 | 11.1 | 6.4 | 3.1 | 0.2 |
| MELO3C024515 | 9677903 | 9680982 | splicing factor U2af small subunit B-like | 1 | 68.1 | 82.1 | 86.2 | 99.5 | 45.5 |
| MELO3C024514 | 9682886 | 9687564 | enolase | 0 | 339.8 | 401.2 | 417.8 | 527.5 | 241.3 |
| MELO3C024513 | 9694722 | 9699966 | glucomannan 4-beta-mannosyltransferase 9-like | 0 | 59.3 | 2.7 | 3.6 | 4.8 | 1.5 |
| MELO3C019311 | 9713031 | 9713408 | glucomannan 4-beta-mannosyltransferase 9-like | 0 | 0.0 | 0.0 | 0.0 | 0.0 | 0.0 |
| MELO3C032937 | 9715540 | 9717395 | glucomannan 4-beta-mannosyltransferase 9-like | 0 | - | - | - | - | - |
| MELO3C024511 | 9738405 | 9740144 | Haloacid dehalogenase-like hydrolase (HAD) superfamily protein | 2 | 0.0 | 0.0 | 0.0 | 0.0 | 0.0 |
| MELO3C024510 | 9741619 | 9745691 | 3-deoxy-d-manno-octulosonic-acid transferase | 3 | 2.1 | 3.4 | 3.5 | 4.1 | 1.0 |
| MELO3C024509 | 9754209 | 9761825 | Pyruvate kinase | 4 | 63.6 | 100.7 | 83.0 | 135.0 | 51.8 |

^1^According to the database Melonet-DB, generated from the cv. Harukei-3.
