# Supplementary figures and images for: Genetic dissection of climacteric fruit ripening in a melon population segregating for ripening behavior
Source: Hortic Res. 2020 Nov 1;7:187. doi: 10.1038/s41438-020-00411-z (PMC7603510; doi:10.1038/s41438-020-00411-z)

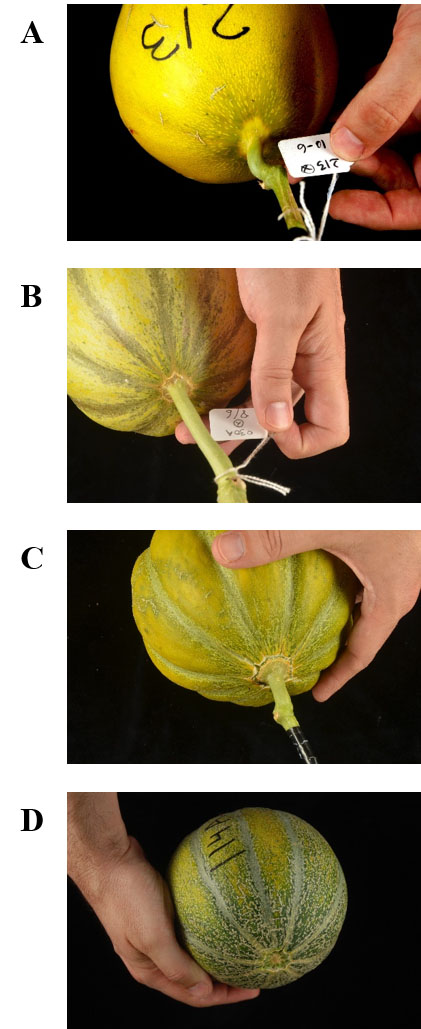

Supplement: Supplementary file 2 — Supplementary Figure 1 [file 41438_2020_411_MOESM2_ESM.jpg]

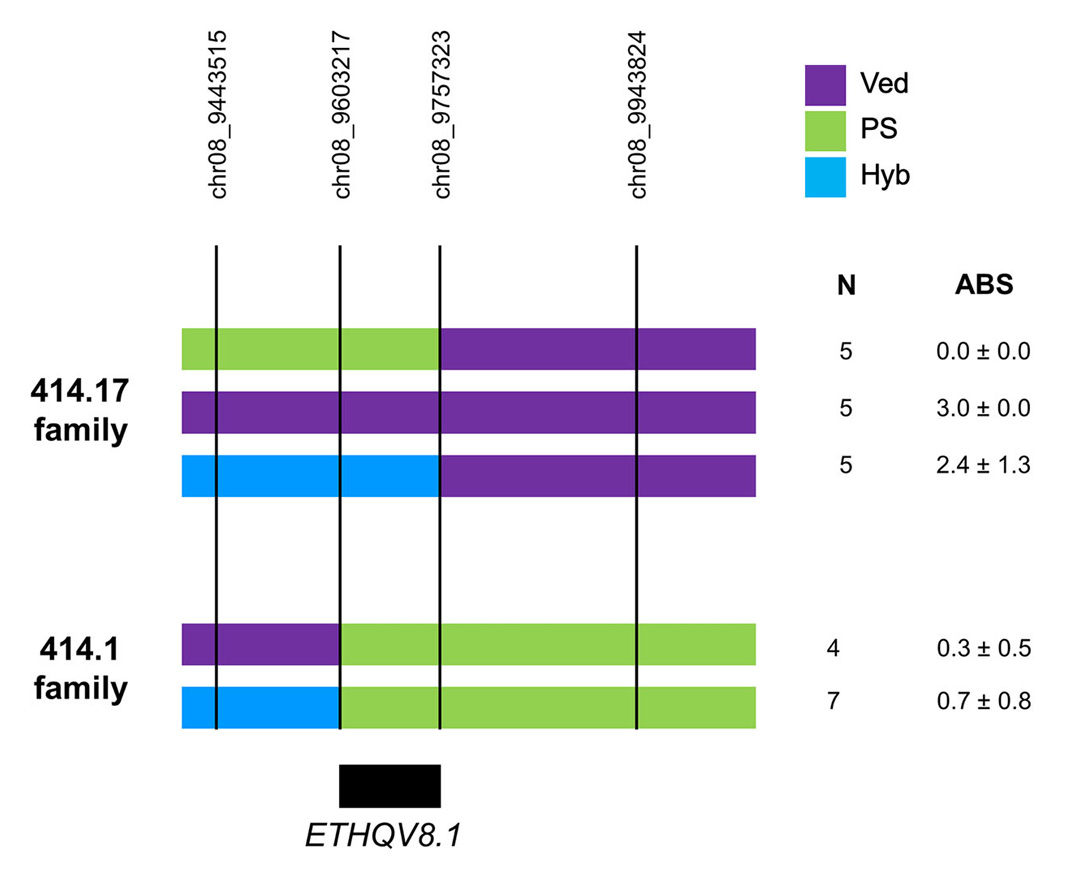

Supplement: Supplementary file 3 — Supplementary Figure 5 [file 41438_2020_411_MOESM3_ESM.jpg]

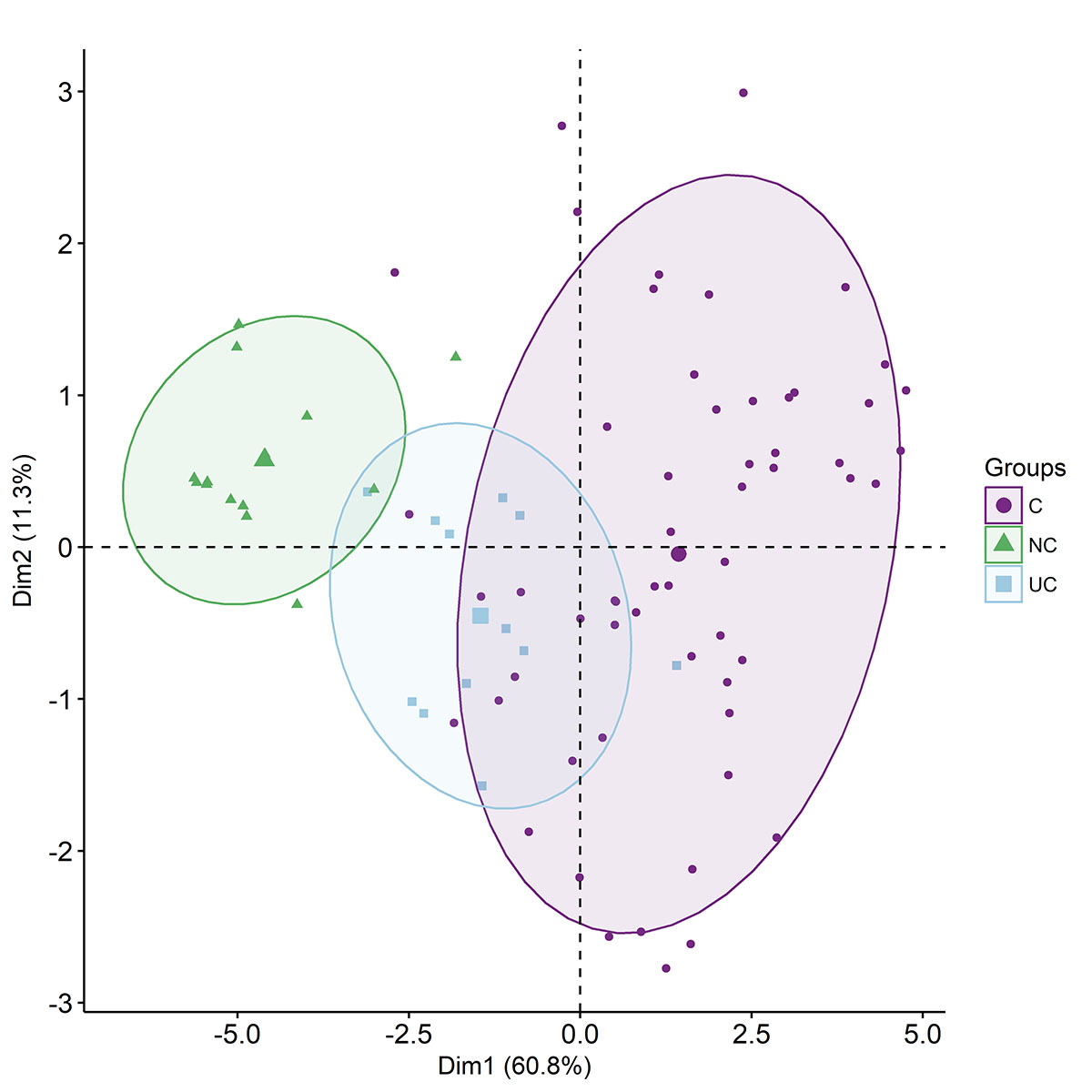

Supplement: Supplementary file 4 — Supplementary Figure 7 [file 41438_2020_411_MOESM4_ESM.jpg]

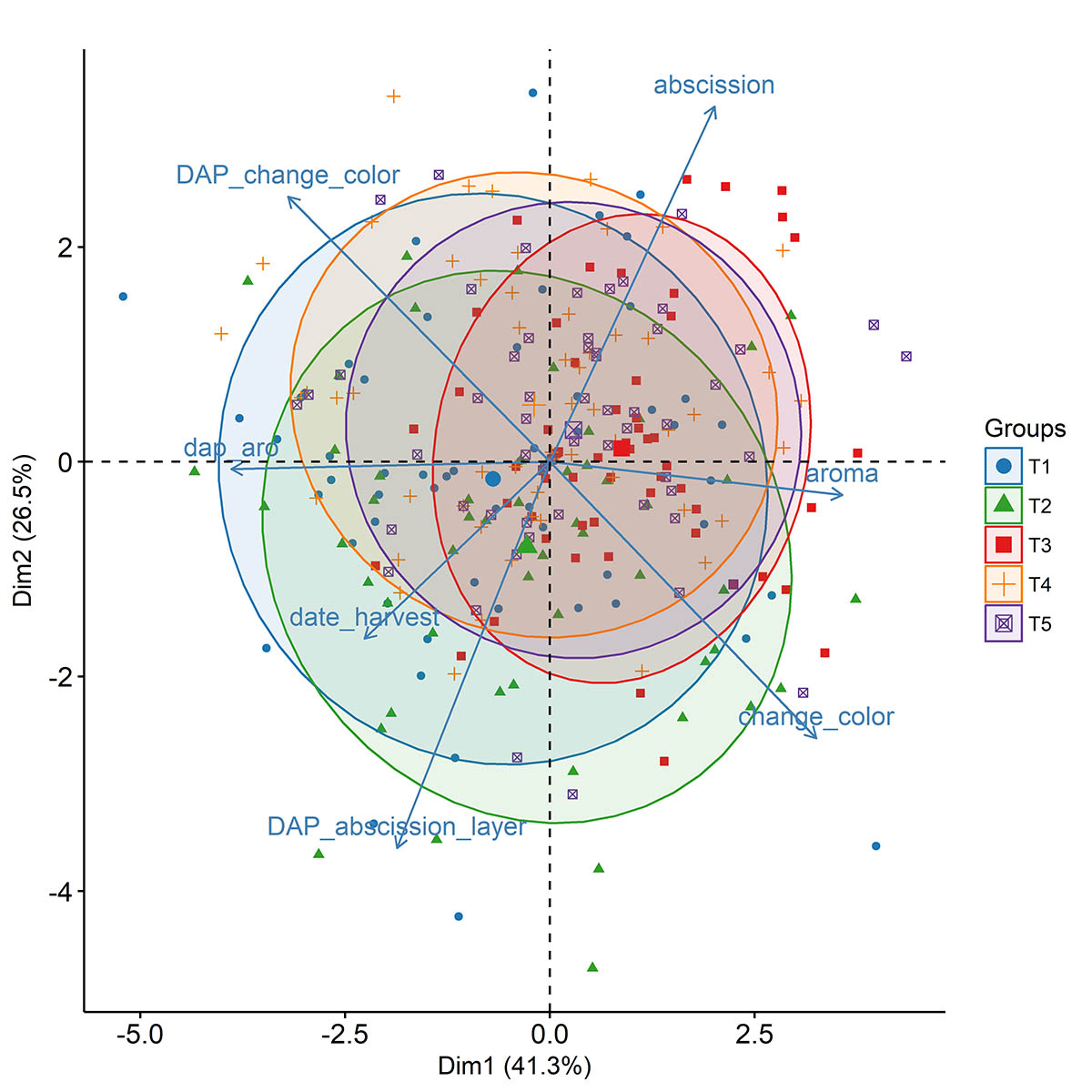

Supplement: Supplementary file 5 — Supplementary Figure 9 [file 41438_2020_411_MOESM5_ESM.jpg]

# CTR1

Tree scale: 0.1

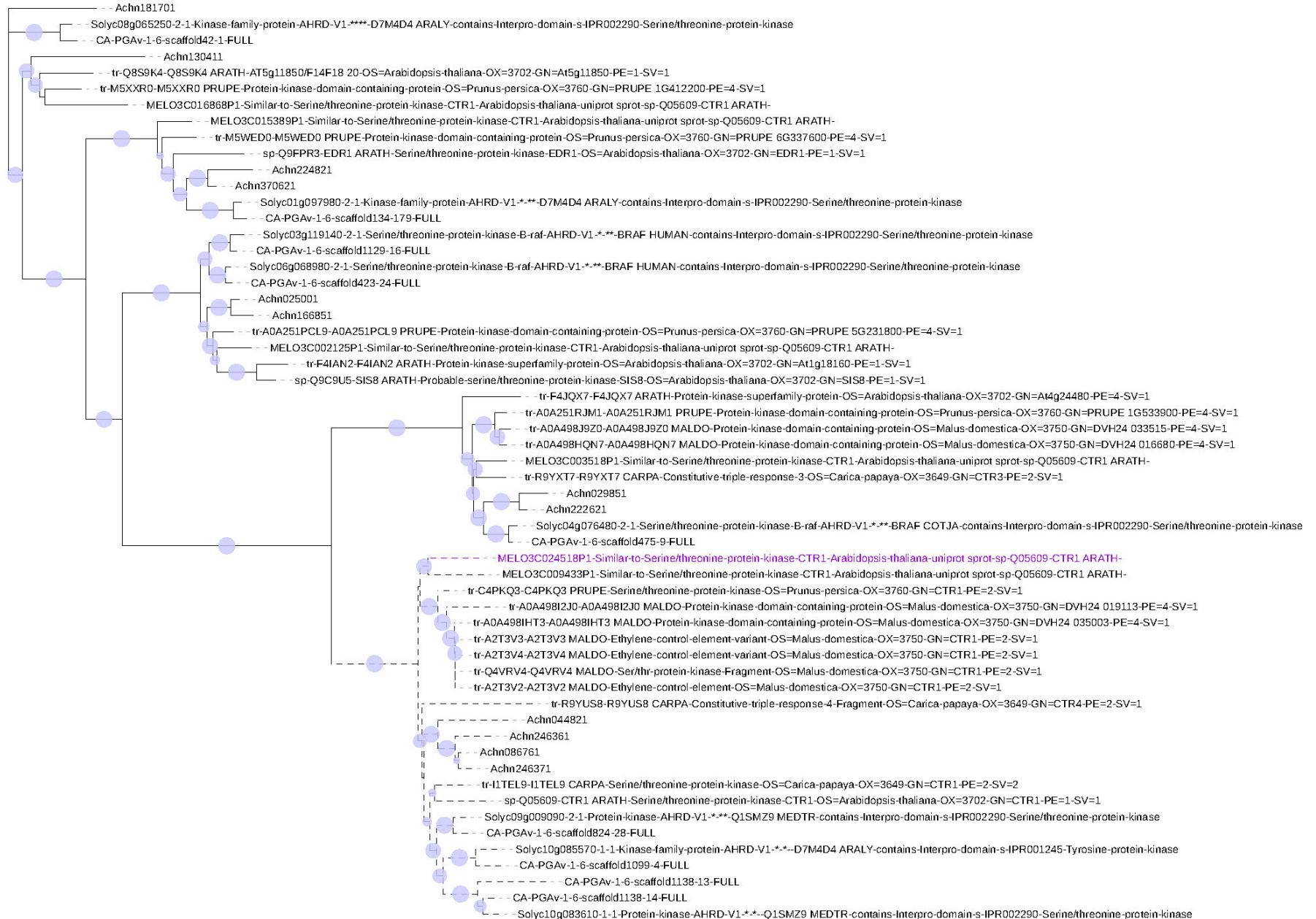

# ROS1

Tree scale: 0.1

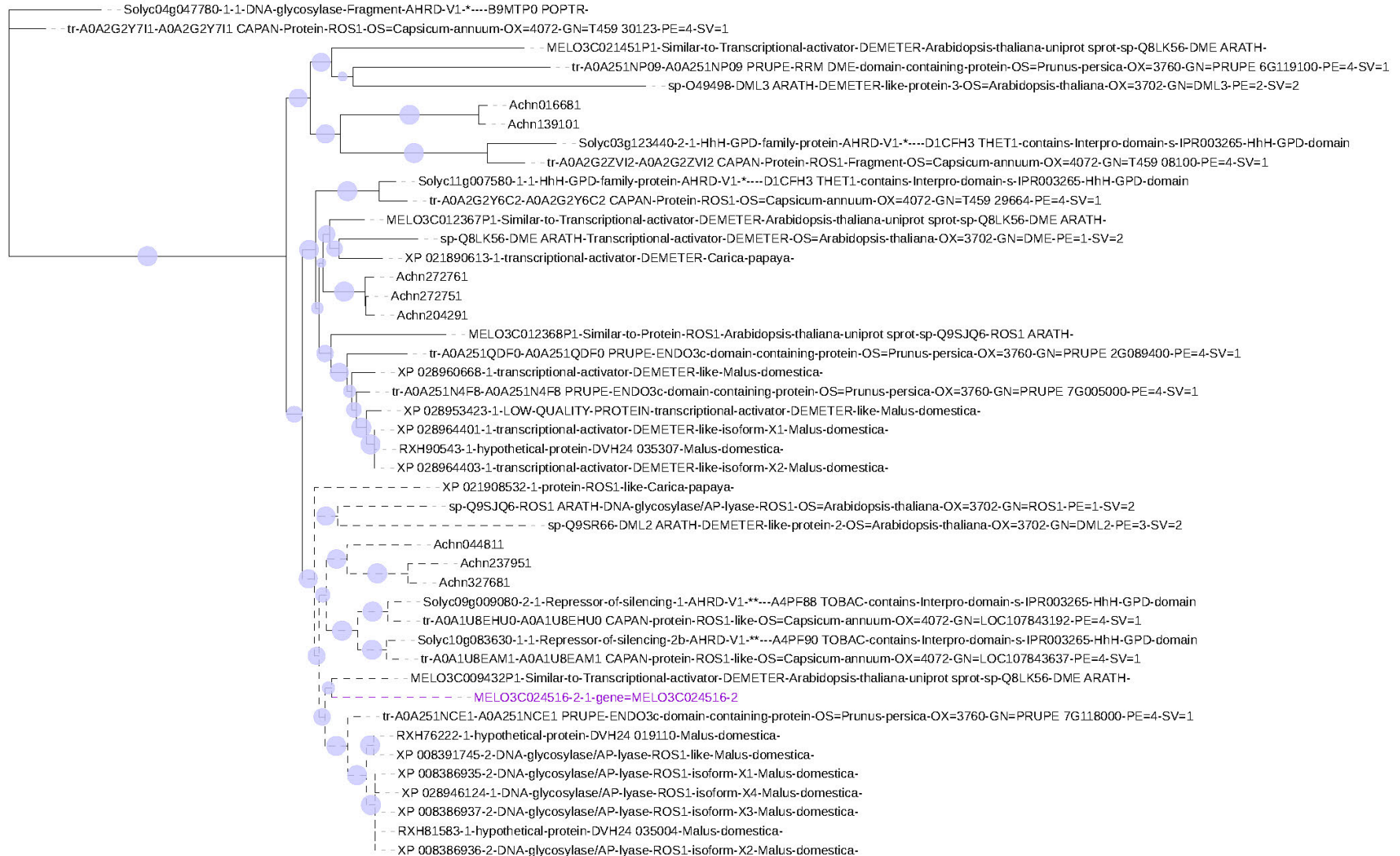

Supplement: Supplementary file 7 — Supplementary Figure 8 [file 41438_2020_411_MOESM7_ESM.pdf]

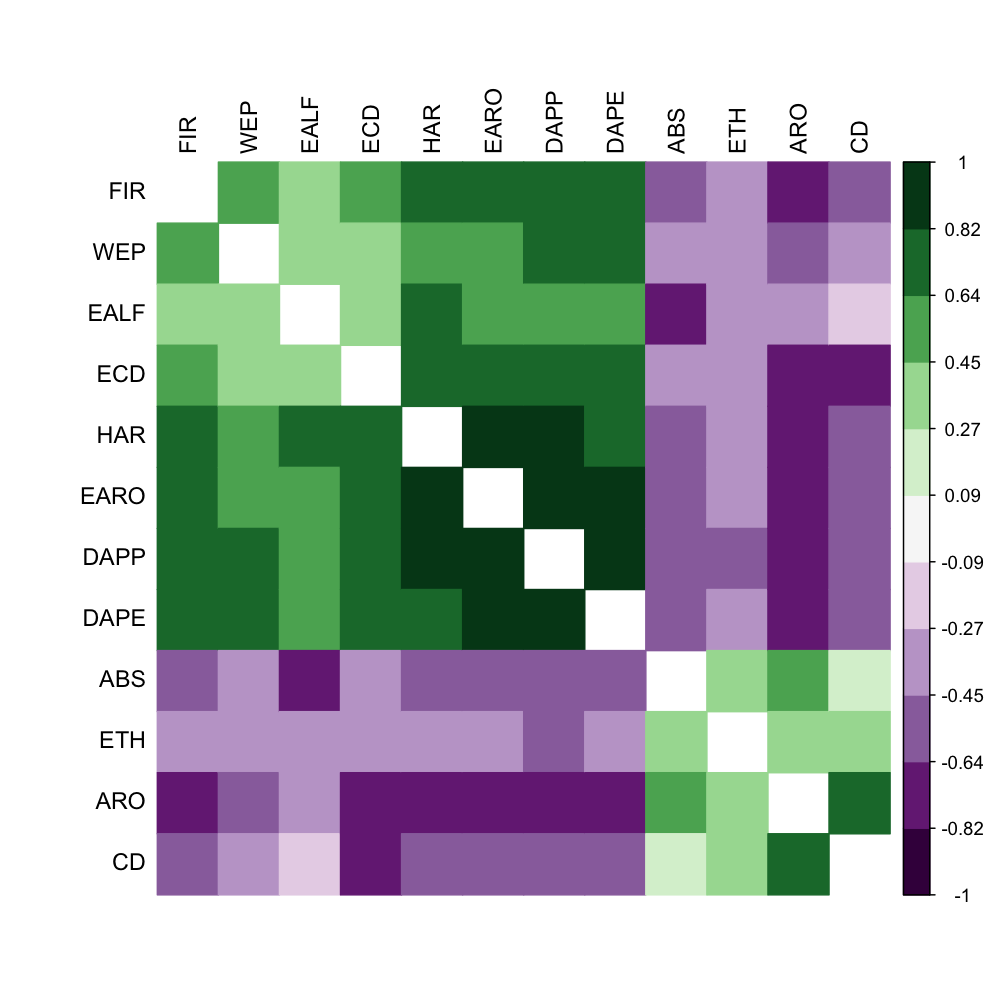

Supplement: Supplementary file 8 — Supplementary Figure 2 [file 41438_2020_411_MOESM8_ESM.png]

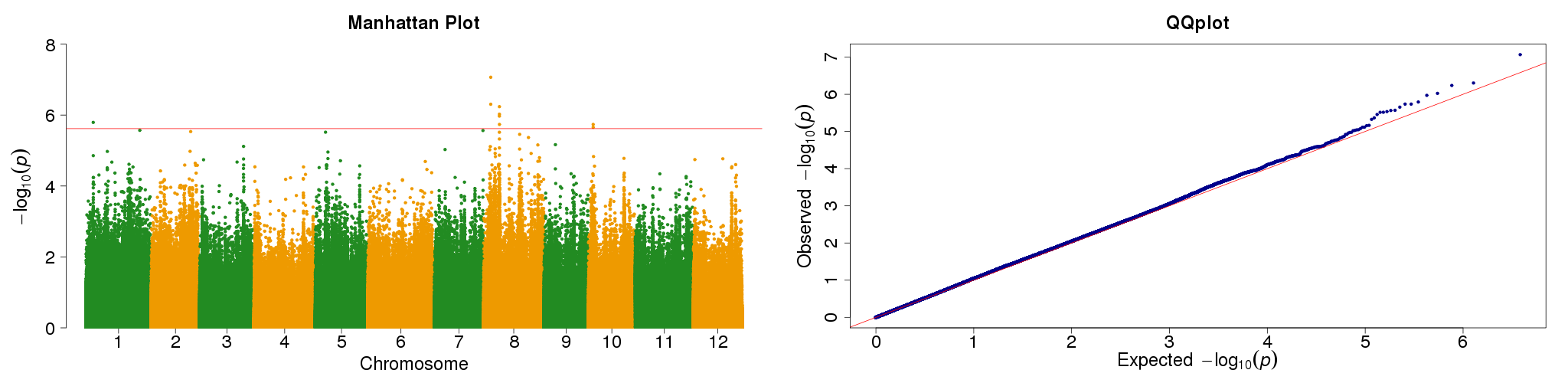

Supplement: Supplementary file 9 — Supplementary Figure 6 [file 41438_2020_411_MOESM9_ESM.png]

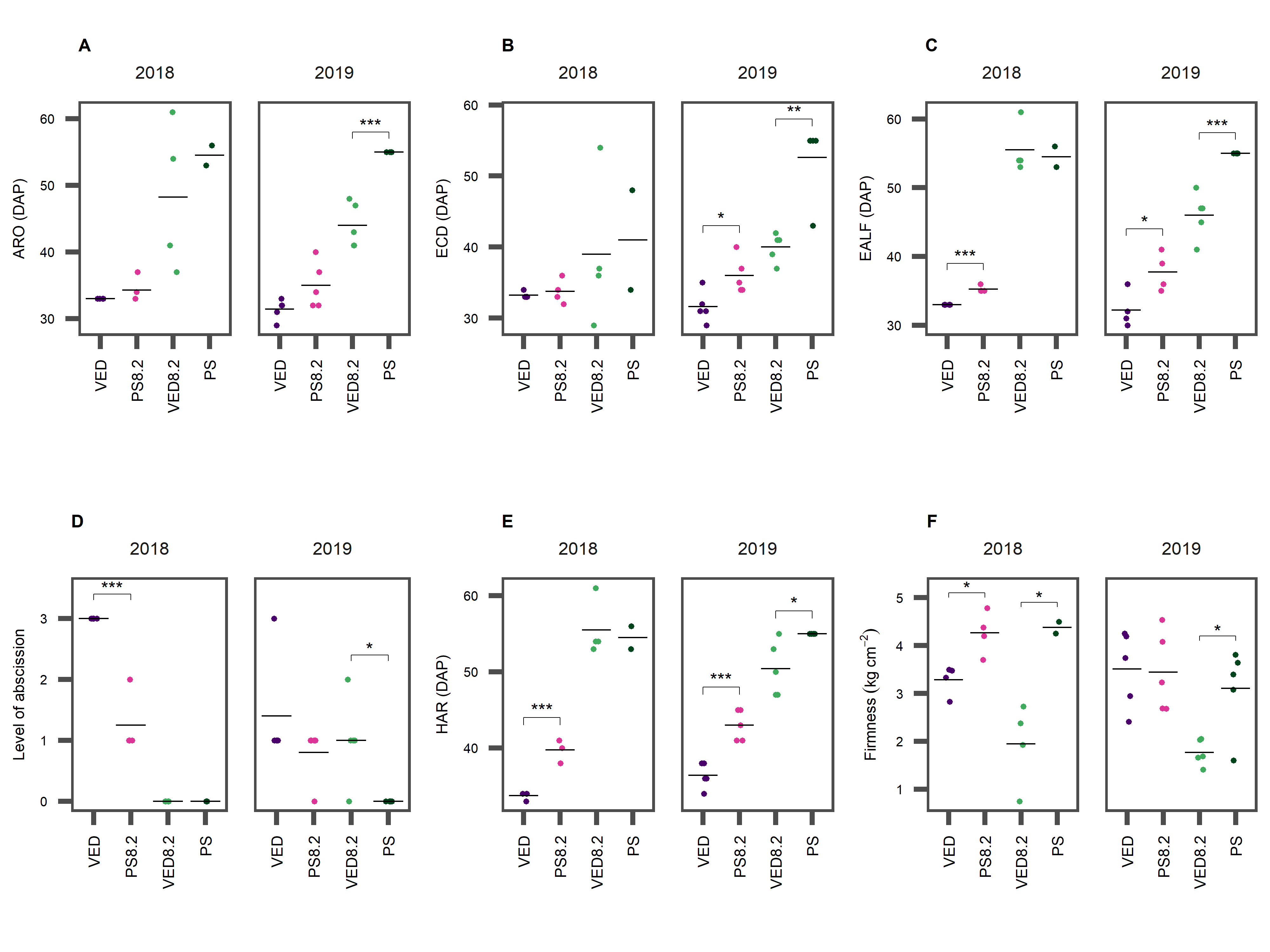

Supplement: Supplementary file 10 — Supplementary Figure 4 [file 41438_2020_411_MOESM10_ESM.tif]
