# Supplementary material for: Genetic dissection of climacteric fruit ripening in a melon population segregating for ripening behavior
Source: Hortic Res. 2020 Nov 1;7:187. doi: 10.1038/s41438-020-00411-z (PMC7603510; doi:10.1038/s41438-020-00411-z)
Supplement: Supplementary file 6 — Supplementary Figure 3 [file 41438_2020_411_MOESM6_ESM.pdf]

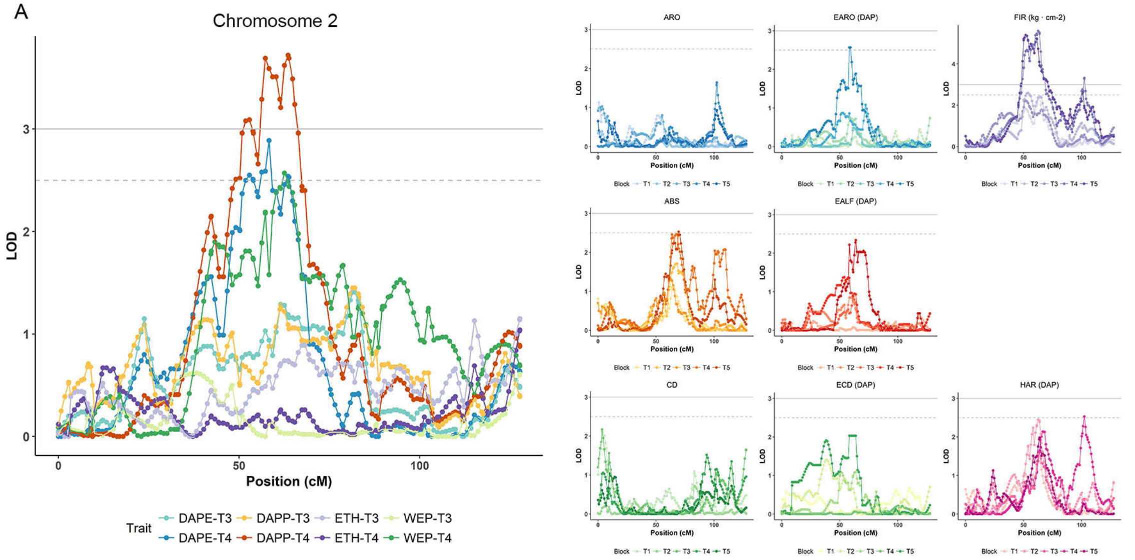
A
Chromosome 2
LOD
Position (cM)
Trait
DAPE-T3
DAPP-T3
ETH-T3
WEP-T3
DAPE-T4
DAPP-T4
ETH-T4
WEP-T4
ARO
EARO (DAP)
FIR (kg · cm-2)
ABS
EALF (DAP)
CD
ECD (DAP)
HAR (DAP)
Block T1 T2 T3 T4 T5

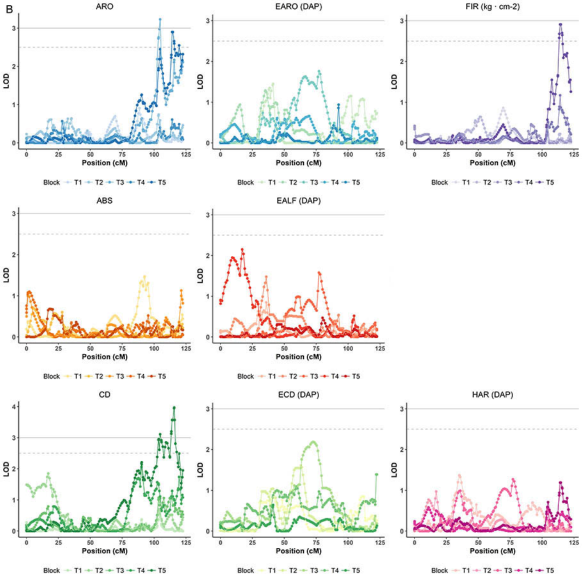

B
ARO
EARO (DAP)
FIR (kg · cm-2)
ABS
EALF (DAP)
CD
ECD (DAP)
HAR (DAP)
LOD
Position (cM)
Block T1 T2 T3 T4 T5

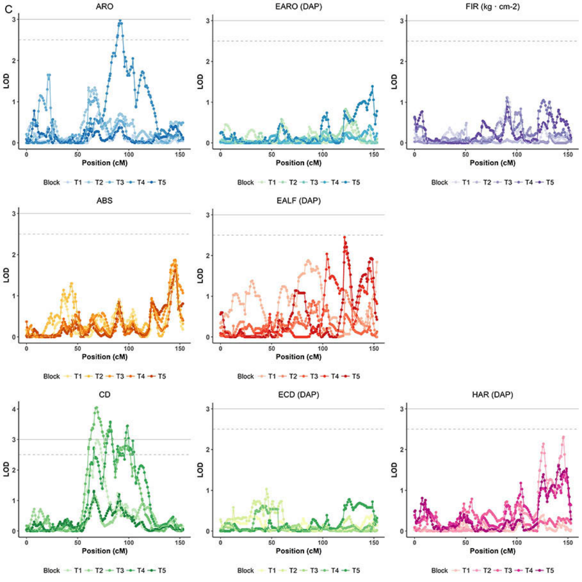
C
ARO
EARO (DAP)
FIR (kg · cm-2)
ABS
EALF (DAP)
CD
ECD (DAP)
HAR (DAP)
LOD
Position (cM)
Block T1 T2 T3 T4 T5

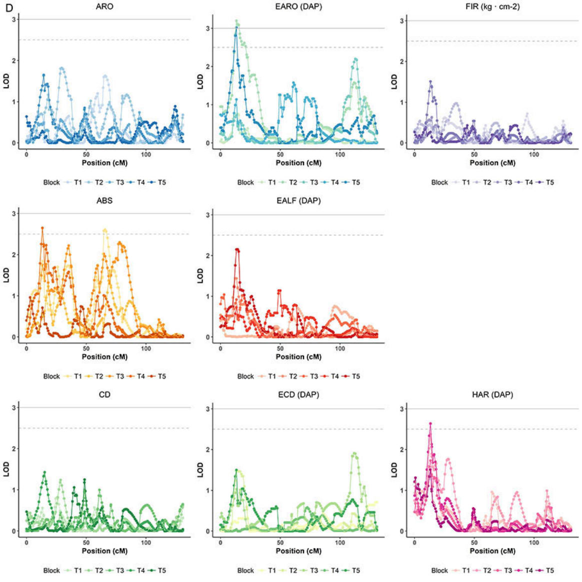
D
ARO
EARO (DAP)
FIR (kg · cm-2)
ABS
EALF (DAP)
CD
ECD (DAP)
HAR (DAP)
LOD
Position (cM)
Block T1 T2 T3 T4 T5

E

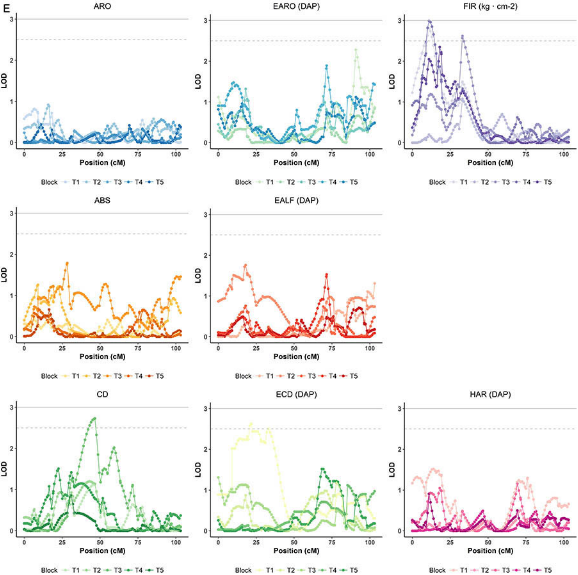
ARO
EARO (DAP)
FIR (kg · cm-2)
ABS
EALF (DAP)
CD
ECD (DAP)
HAR (DAP)
LOD
Position (cM)
Block T1 T2 T3 T4 T5

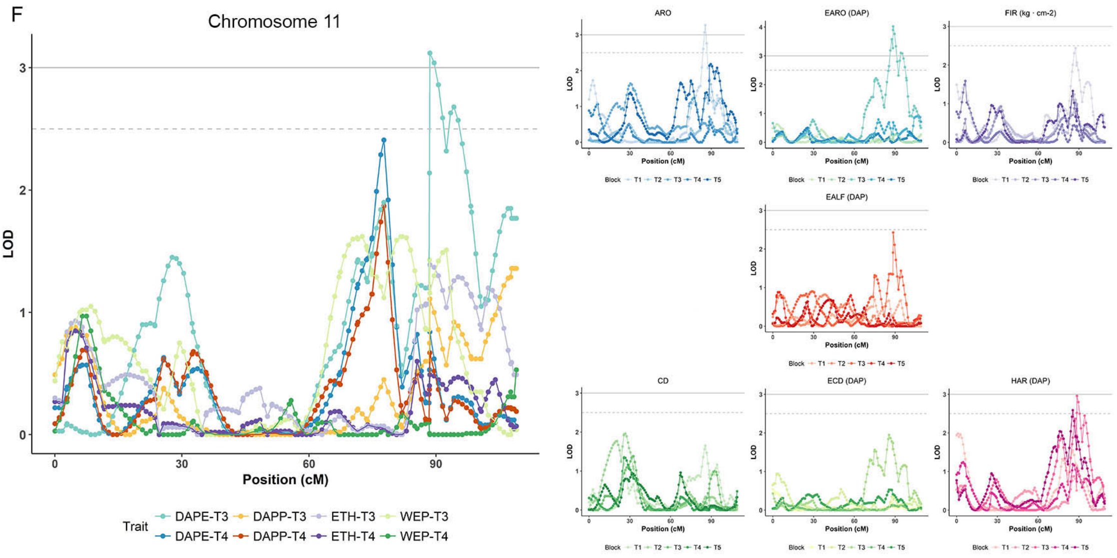
F
Chromosome 11
LOD
Position (cM)
Trait
DAPE-T3
DAPP-T3
ETH-T3
WEP-T3
DAPE-T4
DAPP-T4
ETH-T4
WEP-T4
ARO
EARO (DAP)
FIR (kg · cm-2)
EALF (DAP)
CD
ECD (DAP)
HAR (DAP)
Block
T1
T2
T3
T4
T5
